# Injectable Amoxicillin Versus Injectable Ampicillin Plus Gentamicin in the Treatment of Severe Pneumonia in Children Aged 2 to 59 Months: Protocol for an Open-Label Randomized Controlled Trial

**DOI:** 10.2196/17735

**Published:** 2020-11-02

**Authors:** Lubaba Shahrin, Mohammod Jobayer Chisti, Abu Sadat Mohammad Sayeem Bin Shahid, Abu Sayem Mirza Mohammad Hasibur Rahman, Md. Zahidul Islam, Farzana Afroze, Sayeeda Huq, Tahmeed Ahmed

**Affiliations:** 1 International Centre for Diarrheal Disease Research, Bangladesh Dhaka Bangladesh

**Keywords:** severe pneumonia, treatment failure, amoxicillin, children, randomized controlled trial, Bangladesh

## Abstract

**Background:**

Pneumonia causes about 0.9 million deaths worldwide each year. The World Health Organization (WHO) guidelines for the standard management of severe pneumonia requires parenteral ampicillin every 6 hours and once-daily parenteral gentamicin for 5 to 7 days. Although this treatment has contributed to the reduction of mortality, it requires nursing interventions every 6 hours for 7 days. Further intervention trials should be conducted to search for alternate antibiotics with better adherence, reduced cost, and reduced hospital stay. Parenteral amoxicillin is an effective alternative to ampicillin, as it has a longer half-life and broader coverage.

**Objective:**

The aim of this clinical trial is to compare the efficacy of a dose of injectable amoxicillin every 12 hours plus a once-daily dose of injectable gentamicin with a dose of injectable ampicillin every 6 hours plus a once-daily dose of injectable gentamicin in children hospitalized for severe pneumonia.

**Methods:**

This randomized, controlled, open-label, noninferiority trial is being conducted in Dhaka Hospital of the International Centre for Diarrheal Disease Research, Bangladesh. A sample size of 308 children with severe pneumonia will give adequate power to this study. Children aged 2 to 59 months are randomized to either intravenous ampicillin or intravenous amoxicillin, plus intravenous gentamicin in both study arms. The monitoring of the patients is carried out according to the WHO protocol for the treatment of severe pneumonia. The primary objective is the rate of treatment failure, defined by the persistence of danger signs of severe pneumonia beyond 48 hours or deterioration within 24 hours of initiation of the therapy. The secondary objectives are (1) improvement in or the resolution of danger signs since enrollment, (2) length of hospital stay, (3) death during hospitalization, and (4) rate of nosocomial infections.

**Results:**

Enrollment in the study started on January 1, 2018, and ended on October 31, 2019. Data entry and analysis are in progress. Findings from the study are expected to be disseminated in October 2020.

**Conclusions:**

Our study's findings will improve compliance with the use of antibiotics that require less frequent doses for the treatment of severe pneumonia.

**Trial Registration:**

ClinicalTrials.gov NCT03369093; https://clinicaltrials.gov/ct2/show/NCT03369093

**International Registered Report Identifier (IRRID):**

DERR1-10.2196/17735

## Introduction

Even after remarkable progress in child survival from 1990 to 2015, pneumonia is still considered the leading cause of death globally in children younger than 5 years [1]. Every year, 150 million children contract pneumonia, and 11 million of them require hospitalization [2]. Pneumonia mortality was 0.9 million in 2015 [3,4], which indicates that despite global economic development, improved nutrition, and effective vaccination, more progress is required in case management [5,6]. Case management is one of the key interventions to reduce death in children with pneumonia [7]. Cost-effective interventions, including effective antibiotics, contribute to better outcomes and compliance [8]. Etiological analysis has shown that bacteria and viruses accounted for 33.7% and 54.5% of infection, respectively, in hospitalized patients with severe pneumonia [9,10].

*Streptococcus pneumoniae* were the most common bacteria (33.9%) in cases in which mortality occurred within 30 days of hospitalization [9]. The World Health Organization (WHO) recommends parenteral ampicillin and gentamicin for 5 to 7 days for the treatment of severe pneumonia in children aged 2 to 59 months [11]. As many developing countries, including Bangladesh, lack enough pediatric hospital beds to accommodate the demand for the 5- to 7-day hospitalization of all children with severe pneumonia, alternative treatment options, such as the day care approach, during recovery from danger signs have been investigated and shown to be effective [12,13]. According to local published data, the rate of treatment failure in parenteral ampicillin and gentamicin was 50% [14]; moreover, hospitalization for 5 to 7 days with nursing interventions every 6 hours incur treatment costs and reduce parental compliance with treatment [15]. To address this limitation, we may need to propose a more convenient antibiotic regimen to reduce the cost of treatment and the duration of hospital stay after the disappearance of danger signs. As an alternative drug to ampicillin, amoxicillin has been shown to have an antimicrobial spectrum and level of activity virtually the same as that of ampicillin, with the added advantages of a longer half-life and an equally potent oral formula [16,17]. A combination of amoxicillin and an aminoglycoside may have a synergistic effect against *Streptococcus* species, which are common pathogens for childhood pneumonia [18-20]. When comparing the local cost of these drugs, parenteral amoxicillin is a third of the price of parenteral ampicillin. With the reduced number of intravenous interventions, we also assume a lower incidence of nosocomial infection and a reduced hospital stay. Thus, this report describes the study design of a clinical trial to compare the efficacy of 2 doses of parenteral amoxicillin plus a single dose of gentamicin to 4 doses of parenteral ampicillin plus a single dose of gentamicin in treating children aged 2 to 59 months with WHO-classified severe pneumonia.

## Methods

### Ethics and Research Approval

**Figure 1:**
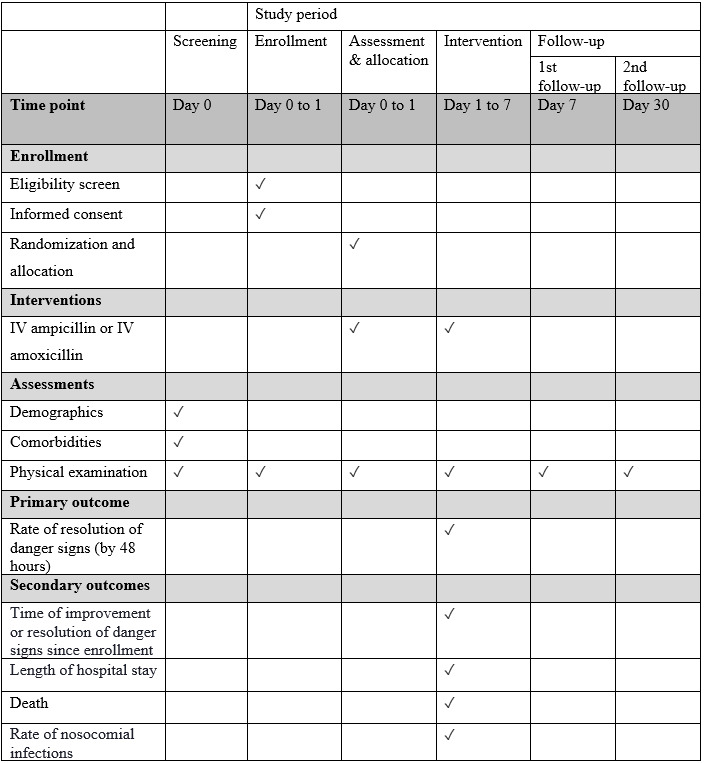
Standard Protocol Items: Recommendation for Interventional Trials (SPIRIT) figure showing the schedule of enrollment, interventions, and assessments. IV: intravenous.

This study was reviewed and approved by the institutional review board at the International Centre for Diarrhoeal Disease Research, Bangladesh (icddr,b) (approval No. PR-17061, version 4.0, dated July 4, 2018). The trial was registered at ClinicalTrials.gov (NCT03369093) on December 11, 2017. To monitor the overall activity of this trial, a data safety and monitoring board (DSMB) was constituted, comprising members of Bangabandhu Sheikh Mujib Medical University, Dhaka University, and the Ethical Review Committee of icddr,b. Results of this study will be disseminated by publication in a peer-reviewed scientific journal and presented at relevant academic conferences and to health policy makers. The study protocol follows the SPIRIT (Standard Protocol Items: Recommendation for Intervention Trials) guidance for protocol reporting (see Figure 1 and Multimedia Appendices 1 and 2).

The legal guardian of the study participants must personally sign and date the approved version of the informed consent form before any trial-specific procedures are performed (see Multimedia Appendix 3). Written and verbal versions of the participant information and informed consent are presented to the participants and their parent or legal guardian, detailing no less than the exact nature of the trial, what it involves for the participant, the implications and constraints of the protocol, the known side effects, and any risks involved in taking part. It is clearly stated that the participant’s parent or legal guardian is free to withdraw from the trial at any time for any reason without prejudice to future care, without affecting their legal rights, and with no obligation to give the reason for withdrawal. A brief follow-up visit plan is also explained during the consent process.

### Study Design and Settings

This is a single-center, randomized, controlled, open-label, noninferiority trial. A noninferiority design was chosen to evaluate if there is any difference in treatment failure among ampicillin and amoxicillin. The primary objective is to compare the efficacy of 2 doses of injectable amoxicillin plus a single dose of injectable gentamicin to 4 doses of injectable ampicillin plus a single dose of injectable gentamicin in the management of children aged 2 to 59 months hospitalized with severe pneumonia. Efficacy of the 2 drugs will be measured by treatment failure in response to therapy, defined by persistence of any danger signs of severe pneumonia beyond 48 hours or deterioration within 24 hours of initiation of therapy (development of any new danger signs or clinical signs of respiratory failure, development of severe sepsis or meningitis, or radiological deterioration). The secondary objectives are (1) improvement or resolution of danger signs since enrollment, (2) length of hospital stay, (3) death during hospitalization, and (4) rate of nosocomial infections. To determine improvement, clinical signs, including hypoxemia (oxygen saturation by pulse oximetry [SpO_2_] of <90% in room air) [21], grunting, convulsion, abnormal mentation, inability to feed or drink, very severe lower chest wall indrawing, and the normalization of fast breathing, will be monitored from enrollment to discharge.

This study is being conducted at the Dhaka Hospital of icddr,b, Dhaka, Bangladesh. This is the largest diarrheal disease hospital in the world, located in the capital of Bangladesh. In 2019, it provided treatment to over 166,000 diarrheal patients with or without associated complications, including pneumonia, sepsis, malnutrition, or complications related to diarrhea. Details of the settings are described elsewhere [22]. The vast majority of the patients come from lower socioeconomic backgrounds from urban and periurban localities, including slums. This hospital is well equipped, with a modernized critical care unit for critically sick children. All the essential laboratory investigations are available in the International Organization of Standardization (ISO)–accredited laboratory facility (ISO 15190:2003) situated on the same premises. All treatment and lodging in the hospital are provided free of cost.

### Study Participants

Children aged 2 to 59 months are eligible for study enrollment upon meeting clinical criteria of severe pneumonia, as defined by the WHO classification updated in 2014 [6,20].

Those coughing or having difficulty breathing are screened by the study staff for eligibility. All screened patients are allotted a unique identification number and interviewed about contact information and demographics. Eligible children are enrolled in the study upon meeting the case definition of severe pneumonia, with cough or difficult breathing plus at least two of the following: (1) central cyanosis or SpO_2_ of <90%, (2) severe respiratory distress (eg, grunting, very severe chest indrawing), or (3) signs of pneumonia with a general danger sign (eg, inability to breastfeed or drink, lethargy or unconsciousness, convulsion).

To tackle differential misclassification, the study enrollment process is witnessed by a nonstudy hospital care provider. Once eligibility is confirmed, the randomization envelope is opened in the presence of a nonresearch physician.

We exclude children who have been on antibiotic therapy at home or in the hospital for at least 48 hours before coming to the hospital or with a known congenital or chromosomal anomaly (eg, congenital heart disease, laryngomalacia, cleft lip, cleft palate, trisomy 21). We also do not recruit children who present with a life-threatening condition that requires immediate assisted ventilation or referral to a higher facility for critical serum creatinine values.

### Randomization

The allocation ratio is 1:1 with a parallel-group enrollment. The permuted block randomization technique is applied. The sequence of randomization was prepared before the commencement of the study by an independent statistician at icddr,b unrelated to this clinical trial. Randomization is performed by computer-generated random numbers with a prefixed block number unknown to the researcher. For blinding of the treatment arms, randomization numbers are provided to the study investigators in sequentially numbered, sealed, opaque envelopes containing the name of the treatment on a card inside the envelope. The study physician opens the subsequent numbered envelope in the presence of another nonstudy physician of the intensive care unit (ICU) or general ward when a participant has formally entered into the trial after written informed consent from the parents or caregivers. After treatment allocation, the child is assigned the allocated antibiotics by the study physician or the study nurses. The enrolled patient is randomized in either the WHO-recommended ampicillin plus gentamicin arm or the amoxicillin plus gentamicin arm.

### Treatment Procedures

**Figure 2:**
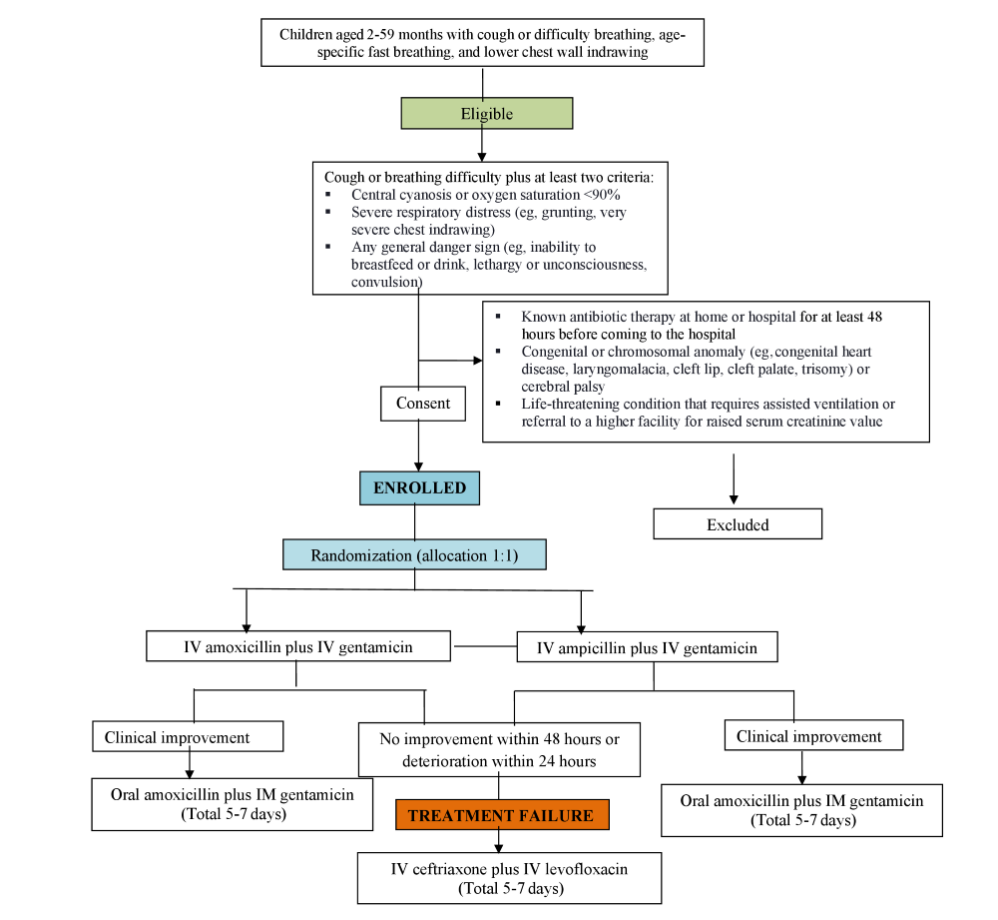
Flowchart for the proposed randomized controlled trial. IM: intramuscular; IV: intravenous.

Hospital standard-of-care management initiates as soon as a patient is admitted. However, the study-specific antibiotic therapy starts following enrollment. The flowchart for the study design is illustrated in Figure 2.

In the ampicillin arm, the patient receives a 50-mg/kg dose of intravenous (IV) ampicillin every 6 hours and a 7.5-mg/kg dose of IV gentamicin once daily for 5 to 7 days. In the intervention arm (amoxicillin arm), the patient receives a 40-mg/kg dose of IV amoxicillin every 12 hours and a 7.5-mg/kg dose of IV gentamicin once daily for 5 to 7 days. In both arms, duration of therapy depends on the patient’s clinical improvement and the clinical judgement of the attending physicians. Children in both regimens require an IV cannula kept in place for the administration of the medication. It is expected that in the absence of complications, clinical improvement should be present [21]. After improvement or resolution of danger signs, when the children can take oral feeding, parenteral ampicillin or amoxicillin is switched to oral amoxicillin at a dose of 40 mg/kg every 12 hours with ongoing intramuscular gentamicin. Children showing subsequent improvement [21] with resolution of all danger signs are advised for discharge under ambulatory medical support, where they receive the remaining doses of intramuscular gentamicin to accomplish the 5- to 7-day course of antibiotic therapy. If any participants refuse the ambulatory medical support due to its far distance, they complete the treatment locally.

At enrollment, oxygen saturation is measured using a pulse oximeter (N-560; Nellcor Puritan Bennett Inc) with a probe on a finger or toe when the child breathes in room air. Oxygen saturation of <90% in room air is defined as hypoxemia, which indicates the need for oxygen therapy. Participants with grunting or hypoxemia are immediately put on bubble continuous positive airway pressure oxygen therapy, following the standard practice of the hospital [14], and kept in the ICU. Participants having feeding difficulty in the form of being unable to suck or swallow, experiencing frequent vomiting (>3 episodes in an hour), or having a respiratory rate of over 70 breaths per minute are started on nasogastric feeding until resolution of symptoms. The management of severe acute malnutrition and related complications is carried out according to standard hospital practice [23,24]. Likewise, the assessment of dehydration and the management of complications is carried out according to hospital protocol [25,26]. The study participants’ care is provided by dedicated study physicians and study nurses by following standard operating procedures. To ensure the uniformity of care, regular training and surprise evaluations are carried out by the trial investigators.

### Data Collection

A structured case report form (CRF) was pretested on 5 pilot patients before formal enrollment began. The CRF is compliant with good clinical practice. Baseline data, demographic and social information (age, sex, religion, parental age, parental occupation, gestational age, weight, drinking water source and sanitation, fuel use, number of rooms in the house, etc), detailed clinical examination data, vital signs including pulse oximetry, and anthropometric measurements are collected and recorded in the CRF. Information is collected about a child’s feeding practices, such as their history of breastfeeding, infant formula, or other complementary feeding practices, as well as immunization status, family history of tuberculosis, recent respiratory tract infections of any family members, and past history of the child’s pneumonia.

After treatment is initiated, data on a participant’s vital signs, calorie intake, progression of illness, new problems, and treatment are recorded every 4 hours in the CRF until discharge. A study physician performs standardized clinical examinations daily to assess clinical signs of treatment failure or clinical improvement.

### Laboratory Tests

All laboratory investigations are carried out according to standard hospital care, and no additional test is advised for this study purpose. For patients with severe pneumonia and malnutrition, complete blood count, chest radiograph, and serum electrolyte tests are performed, according to hospital policy. A blood culture is performed for febrile children.

All laboratory investigation reports and x-ray films are kept covered in the study office under lock and key. Participants’ medical record forms are preserved electronically as a source document. All the medical records are identified by coded numbers to maintain participants’ confidentially and enable tracking throughout the study. Only authorized study staff have access to the files.

No biological products from patients are stored for future use.

### Discontinuation Procedure

The criteria for discontinuing the allocated interventions, as per the study design (Figure 2), are (1) no improvement of danger signs after 48 hours of starting antibiotic therapy; (2) clinical deterioration of the patient in terms of appearance of hypoxemia, grunting, or respiratory failure requiring mechanical ventilation; (3) septic shock or severe sepsis; or (4) referral to other hospitals for conditions such as acute kidney injury (raised serum creatinine). In such instances, patients are declared treatment failures. The ongoing medications are discontinued and the patient is switched to second-line antibiotics (IV ceftriaxone, 80 mg/kg once daily, plus IV levofloxacin, 10 mg/kg once daily) and continued on these medications for 5 to 7 days based on clinical improvement. Once a patient is declared a treatment failure, a blood culture, chest radiogram, and other investigations are recommended, as per standard care. Regardless, the patient is followed by the study team until discharge.

### Follow-up After Discharge

At discharge, patients are advised to return for follow-up visits at day 7 and day 30. During follow-up visits, although no compensation is provided, patients undergo anthropometric measurement and receive a physician’s consultation, including the update on the previous condition. Nutritional follow-up is provided during this visit, along with a demonstration of the preparation of a healthy diet for the children. Clinical examination findings and history of any intercurrent illnesses are recorded in the CRF. If the participants do not return at their scheduled time, the study staff contact the patient’s legal guardian over the phone. In the reachable cases, we ask about the patient’s health status and the presence of any new illnesses after discharge from the hospital and record the answers accordingly. During discharge and follow-up, caregivers are assured that their children will be provided standard treatment in subsequent visits if they wish to bring their child to this hospital.

### Reporting of Adverse Events

All serious adverse events after initiation of the study intervention are recorded and elaborated upon to the DSMB members within 24 hours of the event. The serious adverse event report includes a structured template of the patient’s detailed medical history, the study intervention, the sequence of events, and management attempts until the outcome of the patient. For patients who are referred, the outcome is collected over the phone. The entire safety outcome is analyzed by intention-to-treat analysis.

### Outcome Measures

#### Primary Outcome Variable

The primary outcome variable is the percentage of children with treatment failure, as determined either by the persistence of danger signs over 48 hours or by the appearance of new danger signs within 24 hours of the study intervention.

#### Secondary Outcome Variable

There are 4 secondary outcome variables: (1) time to resolution of danger signs of severe pneumonia, (2) length of hospital stay, (3) rate of nosocomial infections, and (4) death during or after discharge. The secondary outcome measurement variables are the time (in hours) of disappearance of danger signs, time (in days) to discharge from the acute phase, and rate of suspected nosocomial infections (a nosocomial infection will be diagnosed based on the appearance of new signs of infection, such as fever, cough or respiratory distress, diarrhea, or crying during urination, after 48 hours of admission or within 72 hours of discharge from the hospital).

### Sample Size

This is a noninferiority trial. The main therapeutic effect of the intervention therapy (amoxicillin) is not expected to be unacceptably worse than that of the standard therapy (ampicillin) considering all parameters, such as cost, length of hospital stay, and chance of nosocomial infection. Here, the null hypothesis is p_2_ − p_1_ ≥ δ (inferior), where we assume that the proportion cured in the standard arm will be 50% (denoted as π=0.50) [14]. Even if recovery in the intervention arm is 16% less (δ=0.16) than that of the standard arm (ie, up to 34%), we would consider the intervention therapy to be noninferior considering all possible effects. With 80% power and an α of .025 (1-sided), the estimated minimum sample size per group is 154, or 308 in total. Intention-to-treat analysis will be performed. No inflation was added for the consideration of dropout due to cost and time constraints. The sample size calculation was conducted using Stata 15 (StataCorp) using the Statistical Software Components archive package, also known as the Boston College archive.

### Statistical Analysis

Data analyses will be performed using SPSS (version 20.0 for Windows; IBM Corp), Stata (Statistics and data version 13) and Epi Info (version 7.0; Centers for Disease Control and Prevention). Other statistical software packages may be used as required. Statistical analyses will include descriptive as well as analytical methods. Descriptive analysis will be performed between children enrolled in the amoxicillin arm and the ampicillin arm.

Categorical variables will be compared using the chi-square test, and the Fisher exact test will be applied if the expected frequency in any cell is 5 or less. Parametric continuous variables will be compared using 2-tailed Student *t* tests, and nonparametric data will be compared using the Mann-Whitney U test.

As the secondary outcome variables are time to resolution of danger signs and length of hospital stay, survival analysis will be performed to see the response in each group. For the final analysis, a Cox proportional hazard model will be performed.

Logistic regression analysis will be used for predicting the outcome of a categorical (yes or no) variable over one or more predictor variables. Death is a categorical variable (yes or no) and will be analyzed using bivariate analysis. Afterward, a logistic regression model will be prepared to identify the causative variables. A *P* value of <.05 will be considered statistically significant, and the strength of association will be determined by calculating relative risks and their 95% confidence intervals.

Statistical analysis will be performed on data from all randomized patients in the study on an intention-to-treat basis. No per-protocol analysis or imputation of missing data will be performed for this study. Data from children withdrawn because of failure to respond to the usual treatment of severe pneumonia and voluntary dropouts will be included in the analysis up to the time of withdrawal. A supplementary analysis excluding the children withdrawn may be performed.

## Results

Enrollment into the study started on January 1, 2018, and ended on October 31, 2019. Data entry and analysis are in progress. Findings from the study are expected to be disseminated in October 2020.

## Discussion

### Summary

The purpose of this clinical trial is to determine if parenteral amoxicillin is superior to parenteral ampicillin for treating children with severe pneumonia. The WHO-recommended standard antibiotic treatment is successfully reducing child mortality [4,20], but it requires hospitalization for 5 to 7 days to receive antibiotics at 6-hour intervals [21]. Many developing countries, including Bangladesh, do not have enough pediatric hospital beds to accommodate the demand for admission of all children with severe pneumonia. In resource-limited settings, different treatment options, such as the day care approach, with other antibiotic options have been shown to be equally effective in the treatment of severe pneumonia [12]. Therefore, intervention trials should be conducted to search for alternate antibiotics with better adherence, reduced cost, and reduced hospital stay. Parenteral amoxicillin is an effective alternative to ampicillin, as it has a longer half-life and broader coverage. The main purpose of this randomized controlled trial is to assess whether it would be possible to treat severe pneumonia in a hospital with an antibiotic regimen that requires less frequent doses than the current standard. The data gathered from this study will hopefully be of great interest to health care professionals and researchers who seek to modify the management strategy for severe pneumonia in order to achieve a reduced hospital stay and improved treatment outcome.

According to the WHO, standard management of severe pneumonia requires hospitalization with parenteral antibiotics for at least 5 days, and in the absence of any complications, there should be signs of improvement after 48 hours of successful treatment. If the findings from this study show that there is no difference in treatment failure between the 2 arms, then after the improvement of danger signs and the establishment of oral feeding, parenteral amoxicillin can be switched to oral amoxicillin twice daily and intramuscular gentamicin once daily and continued for 5 to 7 days in an ambulatory setting under close monitoring. This will substantially reduce hospitalization costs, reduce parenteral drug usage, and improve patient compliance.

Our hospital has a dedicated respiratory ward with trained respiratory physicians, which allows us to conduct the research with high vigilance while minimizing biases. We expect that the findings of our clinical trial will improve compliance with the use of antibiotics that require less frequent doses in the treatment of severe pneumonia. In essence, the use of parenteral amoxicillin twice daily is cost-effective and requires less frequent parenteral intervention, which should contribute to reducing the incidence of nosocomial infections in our hospital.

### Study Limitations

This study has three main limitations. First, as this is a single-center trial, study findings may not be generalizable to other settings. Second, this is an open-label trial, and the clinical staff could not be blinded due to the different frequencies of drug administration (eg, ampicillin every 6 hours and amoxicillin every 12 hours). We could have blinded the trial by introducing a placebo, but due to the secondary outcome of nosocomial infections, we omitted that. Third, due to cost constraints, we could not perform nasopharyngeal swab cultures to identify the etiology of the pneumonia and compare it with drug sensitivity.

## Appendix

Multimedia Appendix 1 SPIRIT Checklist.

Multimedia Appendix 2 Final Approved IRB protocol.

Multimedia Appendix 3 Informed consent form (local language Bengali).

Multimedia Appendix 4 External review of the Protocol.

## Abbreviations

CRF: case report form
DSMB: data safety and monitoring board
icddr,b: International Centre for Diarrhoeal Disease Research, Bangladesh
ICU: intensive care unit
ISO: International Organization of Standardization
IV: intravenous
SPIRIT: Standard Protocol Items: Recommendation for Intervention Trials
SpO_2_: oxygen saturation by pulse oximetry
WHO: World Health Organization
