# Supplementary material for: Injectable Amoxicillin Versus Injectable Ampicillin Plus Gentamicin in the Treatment of Severe Pneumonia in Children Aged 2 to 59 Months: Protocol for an Open-Label Randomized Controlled Trial
Source: JMIR Res Protoc. 2020 Nov 2;9(11):e17735. doi: 10.2196/17735 (PMC7669443; doi:10.2196/17735)
Supplement: Multimedia Appendix 2 [file resprot_v9i11e17735_app2.pdf]

icddr,b	**RRC APPLICATION FORM**

RESEARCH PROTOCOL
Number: PR-17061
Version No. 4.00
Version date: 10-08-2017

FINAL APPROVED VERSION

FOR OFFICE USE ONLY

RRC Approval:	☒ Yes	☐ No	Date:16-07-2017
ERC Approval:	☐ Yes	☐ No	Date:23-08-2017
AEEC Approval:	☐ Yes	☐ No	Date:
External IRB Approval	☐ Yes	☐ No	Date:

Name of External IRB:

____

Protocol Title:* (maximum 250 characters including space**)**
Efficacy of two doses of parenteral Amoxicillin plus single dose Gentamicin compared to four doses of parenteral Ampicillin plus single dose Gentamicin in managing children hospitalized with WHO classified severe pneumonia: an open labelled randomized controlled clinical trial

Short Title: (maximum **100** characters including space)
RCT of efficacy of Amoxicillin over Ampicillin on severe pneumonia

Key Words:* severe pneumonia, under five children, clinical trial, efficacy, amoxicillin, ampicillin

Name of the Research Division Hosting the Protocol:*

☐ Health Systems and Population Studies Division (HSPSD)
☒ Nutrition and Clinical Services Division (NCSD)
☐ Infectious Diseases Division (IDD)
☐ Maternal and Child Health Division (MCHD)
☐ Laboratory Sciences and Services Division (LSSD)
☐ Other (specify)

Has the Protocol been Derived from an Activity:* ☒ No ☐ Yes (please provide following information):

Activity No. :
Activity Title:
PI:
Grant No.: Budget Code: Start Date: End Date:

icddr,b Strategic Priority/ Initiative (SP 2015-8):*
(check all that apply)

☐ Reducing maternal and neonatal mortality
☒ Controlling enteric and respiratory infections
☐ Preventing and treating maternal and childhood malnutrition
☐ Detecting and controlling emerging and re-emerging infections
☐ Achieving universal health coverage
☐ Examining the health consequences of climate change
☐ Preventing and treating non-communicable diseases

Research Phase (4 Ds):* (check all that apply**)**

☒ Discovery
☐ Development
☒ Delivery
☒ Evaluation of Delivery

Anticipated Impact of Research:* (check all that apply**)**

☒ Knowledge Production
☐ Capacity Building
☐ Informing Policy
☒ Health and Health Sector Benefits
☒ Economic Benefits

**Which of the Sustainable Development Goal This Protocol Relates to?:* (**check all that apply**)**		
☐ 1. End poverty in all its forms everywhere<br>☐ 2. End hunger, achieve food security and improved nutrition and promote sustainable agriculture<br>☒ 3. Ensure healthy lives and promote well-being for all at all ages<br>☐ 4. Ensure inclusive and equitable quality education and promote lifelong learning opportunities for all<br>☐ 5. Achieve gender equality and empower all women and girls<br>☐ 6. Ensure availability and sustainable management of water and sanitation for all<br>☐ 7. Ensure access to affordable, reliable, sustainable and modern energy for all<br>☐ 8. Promote sustained, inclusive and sustainable economic growth, full and productive employment and decent work for all<br>☐ 9. Build resilient infrastructure, promote inclusive and sustainable industrialization and foster innovation<br>☐ 10. Reduce inequality within and among countries<br>☐ 11. Make cities and human settlements inclusive, safe, resilient and sustainable<br>☐ 12. Ensure sustainable consumption and production patterns<br>☐ 13. Take urgent action to combat climate change and its impacts<br>☐ 14. Conserve and sustainably use the oceans, seas and marine resources for sustainable development<br>☐ 15. Protect, restore and promote sustainable use of terrestrial ecosystems, sustainably manage forests, combat desertification, and halt and reverse land degradation and halt biodiversity loss<br>☐ 16. Promote peaceful and inclusive societies for sustainable development, provide access to justice for all and build effective, accountable and inclusive institutions at all levels<br>☐ 17. Strengthen the means of implementation and revitalize the global partnership for sustainable development		
**Does this Protocol Use the Gender Framework:***<br>(Please visit: http://www.icddrb.net.bd/jahia/Jahia/pid/684 for Gender Alanysis Tool with instructions)	☒ Yes (please see attached gender analysis tool page 60)<br>☐ No	
If 'no' is the response, its reason(s) in brief:		
**Will this Research Specifically Benefit the Disadvantaged (**economically, socially and/or otherwise**):**	☐ Yes<br>☒ No	
**Does this Protocol use Behaviour Change Communication:**	☐ Yes<br>☒ No	
**Principal Investigator (Should be icddr,b staff):***Sex ☒ Female☐ Male<br><br>**Dr. Lubaba Shahrin**<br>(Position, phone no, extension no, cell, and email address**):**<br>Associate Scientist, NCSD & Lead, ARI ward, Dhaka Hospital<br>Telephone no. +8809666771100 Ext 3391, Cell number: +8801754598328, lubabashahrin@icddrb.org<br><br>Do you have ethics certification? ☐ No ☒ Yes (please attach in your CV below)<br>Do you have RBM training certification? ☐ No ☒ Yes (please attach the certificate with CV below)	Primary Scientific Division of the PI<br>NCSD	

**Co-Principal Investigator(s) Internal**: Sex ☐ Female ☒ Male<br><br>**Dr. Mohammod Jobayer Chisti**<br>(Position, phone no, extension no, cell, and email address **):**<br>Senior Scientist, NCSD, & Head, Clinical Research, Hospitals, & Clinical Lead, ICU & Consultant Physician, ARI ward, Dhaka Hospital, icddr,b; Dhaka, Bangladesh.<br>Ext: 2334; Cell phone: +8801749292703; Email: chisti@icddrb.org<br>Cell number: +880 1749 292703; Email: chisti@icddrb.org<br><br>Signature or written consent of Co-PI: ___________________<br>(electronic signature or email or any sort of written consent)<br>[if more than one, please copy and paste this row for additional Co-PIs]<br><br>Do you have ethics certification? ☐ No ☒ Yes (please attach in your CV below)<br>Do you have RBM training certification? ☐ No ☒ Yes (please attach the certificate with CV below)	Primary Scientific Division/ Programme of the Co-PI NCSD<br><br>_____________________________<br>Approval of the Respective Senior Director/ Programme Head<br><br>(Signature)
**Co-Principal Investigator(s) - External:** Sex ☐ Female ☐ Male<br><br>Address (provide full official address, including land phone no(s), extension no. (if any), cell phone number, and email address).<br><br>Signature or written consent of Co-PI: ___________________<br>(electronic signature or email or any sort of written consent)<br>[if more than one, please copy and paste this row for additional Co-PIs]	
**Co-Investigator(s) - Internal:** Sex ☐ Female ☒ Male<br>**Dr. Tahmeed Ahmed**<br>(Position, phone no, extension no, cell, and email address **):**<br>Senior Director, NCSD, icddr,b; 68 Shaheed Tajuddin Ahmed Sarani Mohakhali, Dhaka 1212, Bangladesh; Telephone no. +88 02 984 0526-32, Ext: 2358, Cell number: +880 1678 127723; Email: sayeeda@icddrb.org<br><br>Signature or written consent of Co-I: ___________________<br>(electronic signature or email or any sort of written consent)<br>[if more than one, please copy and paste this row for additional Co-Is<br><br>Do you have ethics certification? ☐ No ☒ Yes (please attach in your CV below)<br>Do you have RBM training certification? ☐ No ☒ Yes (please attach the certificate with CV below)	Primary Scientific Division of the Co-I<br>NCSD<br><br>_____________________________<br>Approval of the Respective Senior Director/ Programme Head<br><br>(Signature)

**Co-Investigator(s) - Internal:** Sex ☒ Female ☐ Male<br>**Dr. Sayeeda Huq**<br>(Position, phone no, extension no, cell, and email address **):**<br>Associate Scientist, NCSD and Physician, Clinical Nutrition Unit, Dhaka Hospital, icddr,b; 68 Shaheed Tajuddin Ahmed Sarani Mohakhali, Dhaka 1212, Bangladesh; Telephone no. +88 02 984 0526-32, Ext: 2358, Cell number: +880 1678 127723; Email: sayeeda@icddrb.org<br><br>Signature or written consent of Co-I: ___________________<br>(electronic signature or email or any sort of written consent)<br>[if more than one, please copy and paste this row for additional Co-Is<br><br>Do you have ethics certification? ☐ No ☒ Yes (please attach in your CV below)<br>Do you have RBM training certification? ☐ No ☒ Yes (please attach the certificate with CV below)	Primary Scientific Division of the Co-I NCSD<br><br>_____________________________<br>Approval of the Respective Senior Director/ Programme Head<br><br>(Signature)
**Co-Investigator(s) - Internal:** Sex ☒ Female ☐ Male<br><br>**Dr Farzana Afroze**<br><br>Senior Medical Officer/Assistant Scientist, NCSD,<br>Cell phone: +8801722363067;email: farzanaafroz@icddrb.org<br><br>Signature or written consent of Co-I: ___________________<br>(electronic signature or email or any sort of written consent)<br>[if more than one, please copy and paste this row for additional Co-Is<br><br>Do you have ethics certification? ☐ No ☒ Yes (please attach in your CV below)<br>Do you have RBM training certification? ☐ No ☐ Yes (please attach the certificate with CV below)	Primary Scientific Division of the Co-I NCSD<br><br>_____________________________<br>Approval of the Respective Senior Director/ Programme Head<br><br>(Signature)
**Co-Investigator(s) - Internal:** Sex ☐ Female ☒ Male<br><br>**Dr. Abu Sadat Mohammad Sayeem Bin Shahid**<br>Assistant Scientist, NCSD, Cell phone:+8801819435289email:sayeem@icddrb.org<br><br>Signature or written consent of Co-I: ___________________<br>(electronic signature or email or any sort of written consent)<br>[if more than one, please copy and paste this row for additional Co-Is<br><br>Do you have ethics certification? ☐ No ☒ Yes (please attach in your CV below)<br>Do you have RBM training certification? ☐ No ☐ Yes (please attach the certificate with CV below)	Primary Scientific Division of the Co-I<br><br>_____________________________<br>Approval of the Respective Senior Director/ Programme Head<br><br>(Signature)

Co-Investigator(s) – External: Sex ☐ Female ☐ Male
Address (provide full official address, including land phone no(s), extension no. (if any), cell phone number, and email address):
Signature or written consent of Co-I: ___________________
(electronic signature or email or any sort of written consent)
[if more than one, please copy and paste this row for additional Co-Is]

**Student Investigator(s) - Internal:** Sex ☐ Female ☐ Male<br><br>(Position, phone no, extension no, cell, and email address **):**<br><br>Signature or written consent of Student Investitor: ___________________<br>(electronic signature or email or any sort of written consent)<br>Have ethics certificate? ☐ No ☐ Yes (If Yes, please attach to your CV below)	Students Affiliation<br><br>______________________________<br>Approval of the Respective Senior Director/ Programme Head<br><br>(Signature)

Student Investigator(s) - External: Sex ☐ Female ☐ Male
Address (provide full official address, including land phone no(s), extension no. (if any), cell phone number, and email address):

Signature or written consent of Student Investigator: ___________________
(electronic signature or email or any sort of written consent)

Collaborating Institute(s): Please provide full official address

Institution # 1

Country	
Contact person	
Department<br>(including Division, Centre, Unit)	
Institution<br>(with official address)	
Directorate<br>(in case of GoB i.e. DGHS)	
Ministry (in case of GoB)	

Institution # 2

Country	
Contact person	
Department<br>(including Division, Centre, Unit)	
Institution<br>(with official address)	
Directorate<br>(in case of GoB i.e. DGHS)	
Ministry (in case of GoB)	

Institution # 3

Country	
Contact person	
Department<br>(including Division, Centre, Unit)	
Institution<br>(with official address)	
Directorate<br>(in case of GoB i.e. DGHS)	
Ministry (in case of GoB)	

Note: If less than or more than three collaborating institutions, please delete or insert blocks as needed.

Contribution by the Members of the Scientific Team:

Members' Name	Contribution								
	Research idea/ concept	Study design	Protocol writing	Respond to external reviewers' comments	Defending at IRB	Developing data collection Tool(s)	Data Collection	Data analysis/ interpretation of results	Manuscript writing
Dr Lubaba Shahrin	☒	☒	☒	☒	☒	☒	☒	☒	☒
Dr Mohammod Jobayer Chisti	☒	☒	☒	☒	☒	☒	☐	☒	☒
Tahmeed Ahmed	☒	☒	☒	☒	☒	☒	☐	☒	☒
Dr Sayeeda Huq	☐	☐	☒	☒	☒	☒	☐	☒	☒
Dr Farzana Afroze	☐	☐	☒	☒	☒	☒	☐	☒	☒
Dr Abu SMSB Shahid	☐	☐	☒	☒	☒	☒	☐	☒	☒
	☐	☐	☐	☐	☐	☐	☐	☐	☐
	☐	☐	☐	☐	☐	☐	☐	☐	☐
	☐	☐	☐	☐	☐	☐	☐	☐	☐
	☐	☐	☐	☐	☐	☐	☐	☐	☐
	☐	☐	☐	☐	☐	☐	☐	☐	☐
	☐	☐	☐	☐	☐	☐	☐	☐	☐
	☐	☐	☐	☐	☐	☐	☐	☐	☐
	☐	☐	☐	☐	☐	☐	☐	☐	☐
	☐	☐	☐	☐	☐	☐	☐	☐	☐
	☐	☐	☐	☐	☐	☐	☐	☐	☐

Study Population: Sex, Age, Special Group and Ethnicity

Research Subject:
- ☒ Human
- ☐ Animal
- ☐ Microorganism
- ☐ Other (specify): _____________

Sex:
- ☒ Male
- ☒ Female
- ☐ Transgender

Age:
- ☒ 0 – 4 years
- ☐ 5 – 10 years
- ☐ 11 – 17 years
- ☐ 18 – 64 years
- ☐ 65 +

Special Group:
- ☐ Pregnant Women
- ☐ Fetuses
- ☐ Prisoners
- ☐ Destitutes
- ☐ Service Providers
- ☐ Cognitively Impaired
- ☐ CSW
- ☐ Expatriates
- ☐ Immigrants
- ☐ Refugee
- ☐ Others (specify): ________________

Ethnicity:
- ☒ No ethnic selection (Bangladeshi)
- ☐ Bangalee
- ☐ Tribal group
- ☐ Other (specify): ________________

NOTE: It is icddr.b's policy to include men, women, children and transgender in its research projects involving participation of humans, unless there is strong justification(s) for their exclusion.

Consent Process: (Check all that apply**)**

- ☒ Written
- ☐ Oral
- ☐ Audio
- ☐ Video
- ☐ None

Language:

- ☒ Bangla
- ☐ English
- ☐ Other (specify: _______________

Project/Study Site: (Check all that apply**)**

- ☐ Chakaria
- ☐ Bandarban
- ☒ Dhaka Hospital
- ☐ Kamalapur Field Site/HDSS
- ☐ Mirpur (Dhaka)
- ☐ Matlab DSS Area
- ☐ Matlab non-DSS Area
- ☐ Matlab Hospital
- ☐ Mirzapur
- ☐ Bianibazar (Sylhet)
- ☐ Kanaighat (Sylhet)
- ☐ Jakigonj (Sylhet)
- ☐ Other community in Dhaka
  Name: __________________________________
- ☐ Other sites in Bangladesh
  Name: __________________________________
- ☐ Multi-national Study
  Name of the country______________________

**Project/Study Type:** (Check all that apply)<br><br>☐ Case Control Study<br>☒ Clinical Trial (Hospital/Clinic/Field)*<br>☐ Community-based Trial/Intervention<br>☐ Cross Sectional Survey<br>☐ Family Follow-up Study<br>☐ Longitudinal Study (cohort or follow-up)<br>☐ Meta-analysis<br>☐ Programme Evaluation<br>☐ Programme (Umbrella Project)<br>☐ Prophylactic Trial<br>☐ Record Review<br>☐ Secondary Data Analysis<br>Protocol No. of Data Source: ____________<br>☐ Surveillance/Monitoring<br>☐ Systematic Review<br>☐ Other (specify): ________________________<br><br>***Note**: International Committee of Medical Journal Editors (ICMJE) defines Clinical Trial as "*Any research project that prospectively assigns human participants to intervention and comparison groups to study the cause-and-effect relationship between a medical intervention and a health outcome*".<br><br>PI of the RRC- and ERC-approved Clinical Trials should provide necessary information to IRB Secretariat (Research Administration) for registration and uploading into relevant websites (usually at the https://register.clinicaltrials.gov/). They should also provide relevant information to the IRB Secretariat in the event of amendment/modification after their approval by RRC and ERC.

**Biological Specimen:**	
a) Will the biological specimen be stored for future use?	☒ Yes ☐ No ☐ Not applicable
b) If the response is 'yes', how long the specimens will be preserved?	____5______ years
c) What types of tests will be carried out with the preserved specimens?	No additional investigation will be carried out for this clinical trial. All the patients and investigations will be continued according to usual hospital treatment protocol. Sample will be preserved for future molecular study.
d) Will the consent be obtained from the study participants for use of the preserved specimen for other initiative(s) unrelated to this study, without their re-consent?	☒ Yes ☐ No ☐ Not applicable
e) Will the specimens be shipped to other country/ countries? If yes, name of institution(s) and country/countries.	☐ Yes ☒ No ☐ Not applicable<br>Name ____________________________________
f) If shipped to another country, will the surplus/unused specimen be returned to icddr,b? If the response is 'no', then the surplus/unused specimen must be destroyed.	☐ Yes ☐ No ☒ Not applicable
g) Who will be the custodian of the specimen at icddr,b?	PI (Dr Lubaba Shahrin)
h) Who will be the custodian of the specimen when shipped outside Bangladesh?	Not applicable
i) Who will be the owner(s) of the specimens?	Icddrb
j) Has a MoU been signed with regards to collection, storage, use and ownership of specimen? If the response is 'yes', please attach a copy of the MoU.. If the response is 'no', appropriate justification should be provided for not signing a MoU.	☐ Yes ☐ No ☒ Not applicable

Proposed Sample Size: 308
Sub-group (Name of subgroup e.g. Men, Women) and Number

Name	Number	Name	Number
(1) Ampicillin group	154	(3)	
(2) Amoxicillin group	154	(4)	
		**Total sample size**	308

Determination of Risk: Does the Research Involve (Check all that apply**)**

- ☐ Human exposure to radioactive agents?
- ☐ Foetal tissue or abortus?
- ☐ Investigational new device?
  Specify: ___________________
- ☐ Existing data available from Co-investigator?
- ☐ Human exposure to infectious agents?
- ☒ Investigational new drug?
- ☐ Existing data available via public archives/sources?
- ☐ Pathological or diagnostic clinical specimen only?
- ☐ Observation of public behaviour?
- ☒ New treatment regime?

	Yes	No
Will the information be recorded in such a manner that study participants can be identified from the information directly or through identifiers linked to the study participants?	☐	☒
Does the research deal with sensitive aspects of the study participants' sexual behaviour, alcohol use or illegal conduct such as drug use?	☐	☒

Could information on study participants, if available to people outside of the research team:

	Yes	No
a) Place them at risk of criminal or civil liability?	☐	☒
b) Damage their financial standing, reputation or employability, or social rejection, or lead to stigma, divorce etc.?	☐	☒

Do you consider this research: (check one)

☐ Greater than minimal risk	☒ No more than minimal risk	☐ Only part of the diagnostic test

Note: Minimal Risk: The probability and the magnitude of the anticipated harm or discomfort to participants is not greater than those ordinarily encountered in daily life or during the performance of routine physical, psychological examinations or tests, e.g. the risk of drawing a small amount of blood from a healthy individual for research purposes is no greater than when the same is performed for routine management of patients.

**Risk Group of Infectious Agent and Use of Recombinant DNA**			
a) Will specimens containing infectious agent be collected?	☒ Yes	☐ No	☐ Not applicable
b) Will the study involve amplification by culture of infectious agents?	☐ Yes	☒ No	☐ Not applicable

c) If response to questions (a) and/or (b) is 'yes', to which Risk Group (RG) does the agent(s) belong? (Please visit http://www.icddrb.net.bd/jahia/Jahia/pid/684 to review list of microorganism by Risk Group)	☒ RG1 ☐ RG2 ☐ RG3 ☐ RG4
d) Does the study involve experiments with recombinant DNA?	☐ Yes ☒ No ☐ Not applicable

Does the study involve any biohazards materials/agents or microorganisms of risk group 2, 3, or 4 (GR2, GR-3 or GR4)?

☐ Yes ☒ No

[If the response is 'yes'] I, (print name of the PI) affirm that we will use the standard icddr,b laboratory procedures for biosafety of the hazardous materials/agents or microorganisms in the conduction of the study.

______________________________ ____________

Signature of the Principal Investigator **Date**

Dissemination Plan: [please explicitly describe the plans for dissemination, including how the research findings would be shared with stakeholders, identifying them if known, and the mechanism to be used; anticipated type of publication (working papers, internal (institutional) publication, international publications, international conferences/seminars/workshops/ agencies. [Check all that are applicable]

**Dissemination type**	**Response**		**Description (if the response is a yes)**
Seminar for icddr,b scientists/ staff	☐ No	☒ Yes	In the Scientific Seminar of centre
Internal publication	☐ No	☐ Yes	
Working paper	☐ No	☐ Yes	
Sharing with GoB (e.g. DGHS/ Ministry, others)	☐ No	☐ Yes	
Sharing with national NGOs	☐ No	☐ Yes	
Presentation at national workshop/ seminar	☐ No	☒ Yes	Presentation in the CME of Bangladesh Pediatric Pulmonology Forum (BPPF)
Presentation at international workshop/ conference	☐ No	☒ Yes	Study findings will be presented in international pediatric association or other pediatric infectious disease congress
Peer-reviewed publication	☐ No	☒ Yes	Aim to publish as Original Article in peer reviewed journal
Sharing with international agencies	☐ No	☐ Yes	
Sharing with donors	☐ No	☐ Yes	
Policy brief	☐ No	☐ Yes	
Other			
Other			

Funding:

Is the protocol fully funded?	☒ Yes	☐ No
If the answer is yes, please provide sponsor(s)'s name	1. Bill and Melinda Gates Foundation fund for capacity building	
	2.	
Is the protocol partially funded?	☐ Yes	☒ No
If the answer is yes, please provide sponsor(s)'s name	1.	
	2.	

If fund has not been identified:

Is the proposal being submitted for funding?	☐ Yes	☐ No
If yes, name of the funding agency	1.	
	2.	

**Conflict of interest:**<br>Do any of the participating investigators and/or member(s) of their immediate families have an equity relationship (e.g. stockholder) with the sponsor of the project or manufacturer and/or owner of the test product or device to be studied or serve as a consultant to any of the above?	
☒ No	☐ Yes (please submit a written statement of disclosure to the Executive Director, icddr,b)

Proposed Budget: Attached at appendex

Dates of Proposed Period of Support

(Day, Month, Year - DD/MM/YY)

Beginning Date : 15/09/2017

End Date : 14/09/2018

Cost Required for the Budget Period ($)

Years	Direct Cost	Indirect Cost	Total Cost
**Year-1**	40,085		40,085
**Year-2**			0
**Year-3**			0
**Year-4**			0
**Year-5**			0
**Total**	40,085	0	40,085

Certification by the Principal Investigator:

I certify that the statements herein are true, complete and accurate to the best of my knowledge. I am aware that any false, fictitious, or fraudulent statements or claims may subject me to criminal, civil, or administrative penalties. I agree to accept the responsibility for the scientific conduct of the project and to provide the required progress reports including updating protocol information in the NAVISION if a grant is awarded as a result of this application.

I also certify that I have read icddr,b Data Policies and understand the PIs' responsibilities related to archival and sharing of research data, and will remain fully compliant to the Policies. (Note: The Data Policies can be found here: http://www.icddrb.org/who-we-are/data-policies)

____________ ____________

Signature of PI **Date**

Approval of the Project by the Division Director of the Applicant:

The above-mentioned project has been discussed and reviewed at the Division level.

Dr Tahmeed Ahmed ________________________

Name of the Division Director Signature Date of Approval

Table of Contents

RRC APPLICATION FORM 1
Project Summary 14
Hypothesis to be tested: 15
Specific Aims: 15
Background of the Project including Preliminary Observations: 16
Research Design and Methods 18
Sample Size Calculation and Outcome (Primary and Secondary) Variable(s) 21
Data Analysis 22
Ethical Assurance for Protection of human right........ 23
Data Safety Monitoring Plan (DSMP) 24
Use of Animals 25
Collaborative Arrangements........ 25
Facilities Available 25
Literature Cited........ 26
Budget 26
Other Support 28
Biography of the Investigators 28
Format for Consent Form........ 30
Detailed Budget for the study........42
Check-List........44

☒ Check here if appendix is included

Appendix 1: WHO Guideline of pneumonia [page 46]
Appendix 2: Assessment of dehydration [page 46]
Appendix 3: Diagnosis and management of SAM [page 47]
Appendix 4: Case report form [page 48]
Appendix 5: Gender Analysis Framework [page 60]
Appexdix 6 : Consent Form (English) [page 62]
Consent Form (Bengali) [page 64]

Project Summary
[The summary, within a word limit of 300, should be stand alone and be fully understandable.]

Principal Investigator: Dr Lubaba Shahrin

Research Protocol Title: Efficacy of two doses of parenteral Amoxicillin plus single dose Gentamicin compared to four doses of parenteral Ampicillin plus single dose Gentamicin in managing children hospitalized with WHO classified severe pneumonia: an open labelled randomized controlled clinical trial

Proposed start date: 15 September 2017 Estimated end date: 14 September 2018

Background (brief):

Burden: Pneumonia remains the leading infectious cause of death accounting 920,000 children under five around the world. This means a loss over 2,500 child lives every day, or over 100 every hour. Since 2000, the number of child deaths caused by pneumonia has decreased by 47 per cent. The tremendous progress made is due in part to the rapid roll-out of vaccines, better nutrition and improved care-seeking and treatment for symptoms. However, pneumonia hasn't declined as quickly as other diseases such as malaria (58%), HIV/AIDS (61%) and measles (85%).
Knowledge gap: The Lancet Series on Childhood Pneumonia and Diarrhea has reported that case management is one of the three most effective interventions to reduce pneumonia deaths in children. It is also noted that cost effectiveness of these interventions in national health system needs urgent assessment. It was suggested to find out means to reduce hospital stay without compromising the quality of care.
Relevance: The main purpose of our study is to compare the efficacy of two doses of parenteral Amoxicillin plus single dose Gentamicin compared to four doses of parenteral Ampicillin plus single dose Gentamicin. After 72 hours of treatment injectable Amoxicillin or injection Ampicillin will be switched to or replaced by oral Amoxicillin and will be discharged with an advice to attend to Ambulatory Care Unit (ACU) to receive once daily dose of injection Gentamicin for total 5 days. It is anticipated that this modified therapy will reduce hospitalization stay of children with severe pneumonia and would therefore be relevance in countries with resource poor setting. By reducing hospitalization period, this therapy has potentials to reduced hospital acquired infection.

Hypothesis (if any): <u>Rate of treatment failure with two doses of injectable Amoxicillin plus single dose Gentamicin will be not more than that of four doses of injectable Ampicillin plus single dose Gentamicin in the management of children between 2 months to 59 months hospitalized for WHO classified severe pneumonia.</u>

Objectives:

Primary objectives:
To compare the efficacy of two doses of injectable Amoxicillin plus single dose injectable Gentamicin compared to four doses of injectable Ampicillin plus single dose injectable Gentamicin in management of children hospitalized with WHO pneumonia.

Secondary objectives:
To compare time to resolution of danger signs of pneumonia, length of hospital stay, mortality rate, rate of complication requiring mechanical ventilation, and rate of multiorgan or renal or cardiac failure between the two treatment groups.

Methods: This will be an open labelled randomised, controlled trial in (n= 308) of children of 2-59 months of age hospitalized with WHO classified severe pneumonia. Eligible children will be randomized to one of two treatment regimens: (i) two daily doses of parenteral Amoxicillin plus single daily dose of Gentamicin
(ii) four daily doses of parenteral Ampicillin plus single daily dose of Gentamicin.

After 72 hours of treatment with parenteral amoxicillin or parenteral ampicillin, injectable form will be replaced by oral amoxicillin. We will be evaluating the response to therapy in both the arms.

The response of treatment will be measured by clinical improvement in terms of normalization of fast breathing, chest indrawing, fever and improvement of general condition, feeding, and well being. In other terms end point of the study will be treatment failure, which is defined as follow.

Treatment failure:

Treatment failure is defined by the persistence of danger signs of severe pneumonia for >48 hours or deterioration within 24 hours of initiation of therapy or if child needs mechanical ventilation, dies at any time during hospital stay or left the hospital against medical advice.

Danger Sign:

i) Inability to breastfeed or drink
ii) Lethargy or reduced level of consciousness
iii) Convulsions

Outcome measures/variables:

- Primary outcome is treatment failure
- Secondary outcome (i) time of resolution or recovery from pneumonia (ii) danger sign (iii)total hospitalization period for pneumonia

Description of the Research Project

Hypothesis to be tested:

In a hypothesis testing research proposal, briefly mention the hypothesis to be tested and provide the scientific basis of the hypothesis, critically examining the observations leading to the formulation of the hypothesis.

Does this research proposal involve testing of hypothesis: ☐ No ☒ Yes (describe below)

On the basis of the above background, our primary hypothesis is that:
Treatment failure (defined above) with two doses of injectable Amoxicillin plus single dose of injectable Gentamicin will be comparable with four doses of injectable Ampicillin plus single dose of injectable Gentamicin in the management of children under five hospitalized for WHO classified severe pneumonia.

Specific Aims:

Describe the specific objectives of the proposed study. State the specific parameters, gender aspects, biological functions, rates, and processes that will be assessed by specific methods.

Primary objectives:
To compare the efficacy of two doses of injectable Amoxicillin plus single dose injectable Gentamicin compared to four doses of injectable Ampicillin plus single dose injectable Gentamicin in management of children hospitalized with WHO pneumonia.

Secondary objectives:
To compare time to resolution of danger signs of pneumonia, length of hospital stay, mortality rate, rate of complication requiring mechanical ventilation, and rate of multiorgan or renal or cardiac falure between the two treatment groups.

Primary Outcome: Treatment failure which is defined by the persistence of danger signs of severe pneumonia for >48 hours or deterioration within 24 hours of initiation of therapy (development of new danger signs or clinical signs of respiratory failure, or development of severe sepsis/meningitis, or, radiological deterioration).
Secondary outcomes:

1. Time to resolution of danger signs of severe pneumonia
2. Length of hospital stay
3. Death during hospitalization

Background of the Project including Preliminary Observations:

Provide scientific validity of the hypothesis based on background information of the proposed study and discuss previous works on the research topic, including information on sex, gender and diversity (ethnicity, SES) by citing specific references. Critically analyze available knowledge and discuss the questions and gaps in the knowledge that need to be filled to achieve the proposed aims. If there is no sufficient information on the subject, indicate the need to develop new knowledge.

The World Health Organization (WHO) estimates there are 156 million cases of pneumonia each year in children younger than five years, with as many as 20 million cases severe enough to require hospital admission [1]. In the developed world, the annual incidence of pneumonia is estimated to be 33 per 10,000 in children younger than five years and 14.5 per 10,000 in children 0 to 16 years [2]. Approximately one-half of children younger than five years of age with community-acquired pneumonia (CAP) require hospitalization [3]. In developing countries, respiratory tract infections are not only more prevalent but more severe, accounting for more than 2 million deaths annually; pneumonia is the number one killer of children in these societies [1 4]. Recent study from Dhaka hospital reported a visit of 225 children with severe pneumonia and hypoxemia from 2011 to 2013[5].

The 2008 WHO guidelines for treatment of non severe pneumonia (cough, fever and fast breathing) recommend health workers to provide oral antibiotics for three days at home but urgent referral for hospitalization for parenteral (injectable) antibiotics and other supportive therapy after administration of first dose of antibiotics, if the child has severe pneumonia (cough, fever, fast breathing and lower chest indrawing) or very severe disease (pneumonia with the presence of WHO defined danger signs) [6].Often inability to access a referral facility deprives these children from getting appropriate care. For many families, seeking treatment for their children at a health care facility is often logistically and financially burdensome thus denying them early administration of antibiotics within 48 h that can potentially improve their outcomes [7]. Additionally transport to a distant facility can entail serious delays in effective treatment. Many children with severe pneumonia referred for admission to a hospital could die in transit or reach too sick to be saved [7]. In addition, when hospitalized, the children with severe pneumonia are vulnerable to nosocomial infections in crowded hospital wards and are also at risk of needle-borne infections due to parenteral therapy. Two important studies have addressed such barriers to the recommended treatment of severe pneumonia. The first study was intended to determine whether oral antibiotics are equivalent to injectable antibiotics when both are given in the hospital. This was an open label equivalency study called APPIS (Amoxicillin Penicillin Pneumonia International Study), which was a large multicentre randomized controlled trial comparing injectable penicillin versus oral amoxicillin given for 7 days to children in the hospital [8]. The second study was called "NOSHOTS" (New Outpatient Short-Course Home Oral Therapy for Severe Pneumonia Study) and was a randomized, open-label equivalency trial done at seven study sites in Pakistan and compared initial hospitalization and parenteral ampicillin for 48 h followed by 3 days of oral amoxicillin at home, to 5 days of home-based treatment with oral amoxicillin [9]. NO-SHOTS showed that home treatment with high-dose oral amoxicillin is equivalent to hospital based treatment with parenteral ampicillin in selected children aged 3–59 months with WHO defined severe pneumonia [9]. Later, another study- the MASS study (Multicenter Amoxicillin Severe pneumonia Study) showed that clinical treatment failure and adverse event rates among children with severe pneumonia treated at home with oral amoxicillin did not substantially differ across geographic areas (Bangladesh, Ghana, Vietnam and Egypt) and hence home-based therapy of severe pneumonia could possibly be applied to a wide variety of settings [10]. Thus oral amoxicillin at home has proven clinically efficacious in various settings across the world for treatment of selected children with WHO defined severe pneumonia. The Lancet Series on Childhood Pneumonia and Diarrhoea has reported that case management is one of the three most effective interventions to reduce pneumonia deaths in children but also noted that the cost effectiveness of these interventions in national health systems needs urgent assessment [11].

Antimicrobial selection:

Streptococcus pneumonia is the main pathogen that causes community-acquired pneumonia worldwide, in children under five years (30%-50%) and *Haemophilus influenza (10%-30%)*. Other frequent causes include *Mycoplasma Chlamydia and Legionella* species [12]. Resistance of *S pneumonia* to penicillin and macrolides has been nearly stable in recent years [13] . The introduction of the conjugated pneumococcal vaccine in children has decreased the incidence of invasive penicillin-resistant cases; however, infections with serotypes not affected by the vaccine have increased. Amoxicillin is active against most *S pneumonia* and *H influenza* strains and better absorbed than ampicillin and thus been widely used for treating respiratory infections (including pneumonia) in children [14]. According to WHO pneumonia guideline, children with fast breathing should be treated with Amoxicillin orally at a dosage of 40mg/kg/dose twice daily for five days [15]. In addition children aged 2-59 months with severe pneumonia should be treated with parenteral ampicillin (or penicillin) 100mg/kg/dose 6 hourly and gentamicin 7.5 mg/kg/dose once daily for 3-5 days as a first-line treatment [15].

Antimicrobial properties:

Amoxicillin, an acid stable semi synthetic penicillin shown to be effective against a wide range of infections when given orally and available for intramuscular and intravenous injection. Amoxicillin has an antimicrobial spectrum and level of activity essentially the same as for amoxicillin[16]. Amoxicillin has been shown to have more rapid and complete bactericidal action than ampicillin against E.coli in vitro. Amoxicillin is active at low concentrations against *Staphylococcus aureus* and *epidermidis, Streptococcus pyogens, Diplococcus pneumonia and Streptococcus viridians* and many strains of Streptococcus faecalis. A combination of amoxicillin and an aminoglycoside is synergistic against *Streptococcus* species which are the main pathogens causing childhood pneumonia [16].

Pharmacokinetics:

Peak plasma concentrations and the plasma level profile after intramuscular injection of amoxicillin are dose-related and similar to that after oral administration of an equivalent dosage. Bioavailability is also similar after oral or intramuscular administration. The peak plasma concentration after intravenous injection varies according to the rapidity of the injection but when given by bolus injection (over 3 to 30 seconds) it is 6 to 12 times that attained after the same dose given intramuscularly. A plasma concentration of 5μg/ml is exceeded for about 3 hours after intramuscular and 1 hour after intravenous injection of 500mg amoxicillin, in patients with normal renal function.

Therapeutic Trials*:*

Although the efficacy of oral amoxicillin for several infections in children is well documented, there are relatively few papers on its parenteral use. Nevertheless, published and unpublished data available suggest that amoxicillin administered by the intramuscular and intravenous routes is effective in infections of the urinary and respiratory tracts, and in meningitis and septicaemia caused by a variety of susceptible organisms. Parenteral amoxicillin has been successful in eradicating the original pathogen in about 85% of urinary tract infections caused by Gram-positive or Gram-negative bacteria including those in patients with underlying urinary tract abnormalities in children. At a dosage of 500mg 8-hourly, intramuscular or intravenous amoxicillin has achieved clinical and/or bacteriological responses in 87 to 100% of patients with bronchopneumonia, acute bronchitis or exacerbations of chronic bronchitis although many cases were not proven to be of bacterial origin.

Moreover the injectable amoxicillin is cost effective compare to injection ampicillin. Less manpower is required to administer amoxicillin compared to ampicillin. Both the above facts contributed to less incidence of nosocomial infection. There is not enough research to support this data but our anecdotal observation suggests that the present therapy will have better compliance as you are switching over to oral form.

With the above given facts our aim of conducting this study is to evaluate the efficacy of two doses of parenteral Amoxicillin plus single dose Gentamicin compared to four doses of parenteral Ampicillin plus single dose Gentamicin in managing children under five with severe pneumonia.

Research Design and Methods

Describe the research design and methods and procedures to be used in achieving the specific aims of the research project. If applicable, mention the type of personal protective equipment (PPE), use of aerosol confinement, and the need for the use BSL2 or BSL3 laboratory for different part of the intended research in the methods.. Define the study population with inclusion and exclusion criteria, the sampling design, list the important outcome and exposure variables, describe the data collection methods/tools, and include any follow-up plans if applicable. Justify the scientific validity of the methodological approach (biomedical, social, gender, or environmental).

Also, discuss the limitations and difficulties of the proposed procedures and sufficiently justify the use of them.

Study design: Open label, randomized, controlled intervention study in 308 children between two months to 59 months admitted to the Dhaka Hospital of icddr,b . Children will be randomized to receive (i) Amoxicillin plus single dose Gentamicin (ii) four doses of parenteral Ampicillin plus single dose Gentamicin. After 72 hours of treatment injectable Amoxicillin or injection Ampicillin will be switched to or replaced by oral Amoxicillin.

Eligibility Criteria

Selection of the study population is based on the fact that there should be a sufficient proportion of patient who has true bacterial pneumonia or sepsis. If the study enrols a large proportion of children with bronchiolitis, or viral pneumonia then those with true severe pneumonia will be diluted and the power to see a difference is reduced. Therefore, to yield actual response to therapy we strengthen the entry criteria of enrolment.

Inclusion criteria:

Children more than 2 months to less than 59 months will be enrolled in our study, if they have a history of cough or difficulty in breathing and WHO classified severe pneumonia plus **at least two of the following**:

i) Central cyanosis or oxygen saturation <90% on pulse oxymetry
ii) Severe respiratory distress (e.g. grunting, very severe chest indrawing)
iii) Signs of pneumonia with a general danger sign: inability to breastfeed or drink; lethargy or reduced level of consciousness; convulsion
iv) In addition, some or all of the other signs of pneumonia may be present, such as:
    - Signs of pneumonia [age specific fast breathing(*Annex 1)*]
    - Chest indrawing: lower chest wall indrawing
    - Chest auscultation signs: (any one)
        a) Decreased breath sounds
        b) Bronchial breath sounds
        c) Crackles
        d) Abnormal vocal resonance (decreased over a pleural effusion or empyema, increased over lobar consolidation)
        e) Pleural rub

Exclusion Criteria:

Children with life threatening condition that required immediate assisted ventilation of refer to outside hospital (such as septic shock, cardiac arrest, apnea, respiratory failure etc.) will be excluded from the study. Children with uncorrected cyanotic CHD, hypercapnoea ($PCO_2 > 60$mm of Hg), status asthmaticus and upper-airway obstruction, preterm baby (but not ex-preterm) and those for who inform consent can't be secured from their parents/ care-givers. Care giver presented with any drug container of antibiotics or prescription showing getting antibiotics will be excluded.

Study site and procedure:

This study will be conducted at the Dhaka Hospital of icddr,b, Dhaka, Bangladesh. This hospital is located in Dhaka city, the capital of Bangladesh. It provides care and treatment to around 110,000 diarrheal patients with or without associated complications and with or without other health problems each year. The vast majority of the patients come from poor socio-economic backgrounds from urban and peri-urban Dhaka, the capital city of Bangladesh.

Patients attending this hospital are first assessed by an experienced triage nurse, which include assessment of the type of diarrhoea and degree of dehydration, and presence of other health problems including ALRI/ pneumonia, severe malnutrition, impaired mental status (convulsion), and sepsis. They redirect non-diarrhoeal patients to other appropriate city hospitals in the city. Based on assessment of diarrhoeal patients, those with uncomplicated illness without signs of dehydration are referred to the Out Patient Unit for 4-5 hours of observation, maintenance of hydration using ORS solution, and for provision of health education to the mothers/caregivers. Those with some and severe dehydration but without any associated health problems are admitted to Short Stay Ward (SSW) for correction of dehydration and maintenance of hydration using ORS and/or intravenous fluids, provision of antimicrobial therapy as appropriate, with an average stay of 24 hours. Those with associated complications of diarrhoea and/or associated health problems are referred to a physician for assessing the need for admission to either the Longer Stay Ward (LSW) or Acute Respiratory Ward (ARI) or to the Intensive Care Word (ICU) depending on the clinical severity. The usual associated conditions include difficult respiration, cyanosis, apnoea, hypothermia or hyperthermia, marked lethargy or comatose condition, poor peripheral perfusion not attributable to severe dehydration. Those hospitalised in SSW are assessed by hospital clinicians for possible admission to LSW and further workup and management. After admission to the LSW/ARI/SCU the attending physician obtained medical history, performs thorough clinical examination, makes a problem list including differential diagnoses for each of them, arranges for required laboratory workups, and develops the treatment plan following standard guidelines of the hospital. They also perform bedside procedures such as sampling blood, performing lumber puncture etc. Oxygen saturation is determined by pulse oxymeter when necessary, and blood glucose estimated by bedside Gluco Check machine in nearly all patients admitted to the LSW/ARI /SCU.

Study Interventions:

Children between 2 months to 59 months with severe pneumonia will be studied to evaluate the proportions of treatment failure in two different arms.

We will use sealed envelopes to randomly assign the children to either treatment arms. In first arm children will receive 2 daily doses of parenteral Amoxicillin and single daily dose of parenteral Gentamicin; and in 2nd arm, children will receive 4 daily doses of parenteral Ampicillin and single daily dose of parenteral Gentamicin. After 72 hours of treatment, injection Amoxicillin and injection Ampicillin will be replaced with oral Amoxicillin but injection gentamicin will be continued for total 5 days in once daily dose. During this time patient will be suggested to discharge from hospital and attain to Ambulatory care unit for getting once daily dose of injection Gentamicin. Both the treatment arm will continue for 5 days.

During the study period, children in both study arms will also receive standardized hospital management for diarrhoea and malnutrition *(Annex 2,3)*.

“Treatment failure” will be standardized, based on clinical and monitoring data. Treatment failure is defined by the persistence of danger signs of severe pneumonia for >48 hours or deterioration within 24 hours of initiation of therapy or if child needs mechanical ventilation, dies at any time during hospital stay or left the hospital against medical advice.

During the study period any child from either study group who develops the features of “clinical failure” (any two of three criteria in the definition below) will be managed according to best practice in the clinical context. If children fail any arm of treatment, they should be rescued with second line antibiotic regimen, which is injection ceftriaxone and injection levofloxacin. Hypoxia will be managed by B-CPAP or humidified high flow air / O_2 mix at 2 l/kg/min through nasal cannula.

.

Monitoring of the children with severe pneumonia

The following parameters should be routinely monitored in every child with severe pneumonia. Special attention should be given to child receiving I/V fluid therapy and those with SAM. Such as: Heart rate, respiratory rate, temperature, respiratory pattern including chest recession, lower chest wall in drawing, use of accessory muscles, establishment of oral feeding, liver size, oxygen saturation level, chest auscultation: (rales, rhonchi, bilateral basal crepitation, pleural rub), fluid and calorie intake.

Children on oxygen therapy should have at least 3-4 hourly observations of all the above parameters and children without getting oxygen should be observed at least 2 hourly by the study nurses. All the patients should be evaluated by the physicians at least 12 hourly by the physicians.

Signs of Improvement:

In absence of complications, within 2 days of initiation of treatment there should be signs of improvement

i) breathing rate within normal limit; ii) absence of the lower chest wall in-drawing; iii) less to no fever; iv) improved ability to eat and drink; and v) better oxygen saturation without assistance.

Discharge criteria

- Respiratory distress has resolved
- Oxygen saturation > 90%
- Feeding well
- Able to take oral medication or have completed a course of parenteral antibiotics
- The parents understand the signs of pneumonia, risk factors and when to return

Laboratory investigations:

No additional laboratory investigations will be required to perform this study. Routine management of severe pneumonia will be followed as per the existing pneumonia guideline practiced in Dhaka Hospital. The patients with suspected sepsis will be investigated for source of infection by CBC, Blood culture and chest X-ray. Any patient with severe acute malnutrition (SAM) and cough and/or fast breathing will be investigated for chest X ray and CBC. There will be no incurrence of hospitalization cost to conduct the research.

Respiratory ward Infrastructure:

An independent acute respiratory infection treatment unit was established right at the entrance to the hospital, and started functioning on 6 September, 2009. Patients reporting to Dhaka Hospital were rapidly screened for

respiratory symptoms such as fever, cough, myalgia, headache, chills, fatigue, running nose, sore throat, rapid breathing and difficulty in breathing. Patients with these symptoms were referred to the respiratory triage where they were assessed following the hospital guidelines and relevant information was entered into the hospital's electronic database. Patients with any form of pneumonia (non-severe, severe, and very severe), bronchiolitis, asthma, bronchitis, pleural effusion, empyema, lung abscess, tuberculosis, and any other common respiratory problems are admitted in the ward. Others were referred to the usual diarrhoea triage of the hospital. Sufficient staff provide 24 hour cover from all cadres (doctors, nurses, health workers and auxiliary staff). This facility was provided with all necessary supplies including a portable X- ray machine, Ambu bags, large oxygen cylinders, pulse oxymeters, disposable gloves, disinfectants and medication (e.g. antibiotics and other medicines). Pulse oxymetry is routinely performed to evaluate arterial oxygen saturation, and additional tests were performed if there were clinical indications. Vital signs were monitored, and stool and urine output was measured and recorded 8 hourly. Therapy is decided on the basis of clinical evaluation, results of the laboratory tests, and the hospital guidelines for management of respective respiratory illness.

Ambulatory care Unit:

This is a new set up in Dhaka Hospital and the facility is used to reduce patient hospital stay as well as nosocomial infection rate. This facility is only offered to the patients who have recovered from the acute illness and showing signs of improvement. The patients who live in Dhaka city and able to return to Dhaka hospital are requested to come to receive injectable drugs every morning. Drugs will be provided from hospital free of cost. The total procedure will take less than one hour and patient can go home afterward. By this care the antibiotic course will be completed and hospitalization cost can be minimized.

Sample Size Calculation and Outcome (Primary and Secondary) Variable(s)

Clearly mention your assumptions. List the power and precision desired. Describe the optimal conditions to attain the sample size. Justify the sample size that is deemed sufficient to achieve the specific aims.

Sample size calculation:

[For two-sample comparison of proportion (non-inferiority)]

Null hypothesis: p2-p1≤ δ (inferior), where: pi (entered in command) is the overall proportion of participants expect to experience the outcome if the treatments are non-inferior, and delta is the smallest change in proportions between groups (p2 - p1) which would still be clinically important.
Assumptions:
power = 0.8; alpha = 0.025 (one-sided); pi = 0.50; delta = 0.16
Estimated required sample size (per group) = 154,

Total sample is 308

Primary Outcome: Treatment failure which is defined by the persistence of danger signs of severe pneumonia for >48 hours or deterioration within 24 hours of initiation of therapy (development of new danger signs or clinical signs of respiratory failure, or development of severe sepsis/meningitis, or, radiological deterioration).

Secondary outcomes:

1. Time to resolution of danger signs of severe pneumonia
2. Length of hospital stay
3. Death during hospitalization

Outcome variables

1. The primary outcome variable will be treatment failure (see definition above).
2. The secondary outcome variables will be:
    I. Time to resolution of danger signs of severe pneumonia
    II. Length of hospital stay
    III. Death during hospitalization

Data Analysis

Describe plans for data analysis, including stratification by sex, gender and diversity. Indicate whether data will be analysed by the investigators themselves or by other professionals. Specify what statistical software packages will be used and if the study is blinded, when the code will be opened. For clinical trials, indicate if interim data analysis will be required to determine further course of the study.

Data analysis plan:

Data analyses will be performed using the SPSS, version 20.0 Windows, (SPSS, Chicago, IL) and Epi Info (version 7.0, USD, Stone Mountain, GA). Statistical analyses include descriptive as well as analytical methods. Intension to treat (ITT) analysis will be performed. For categorical variable, the significance of differences will be evaluated comparing Amoxicillin arm with Ampicillin arm by chi-square test or Fisher exact test as appropriate. For continuous variables, for normally distributed data the differences of means will be compared by Student's t-test and for not normally distributed data Mann-Whitney test will be used. A p-value < 0.05 will be considered statistically significant. Strength of association will be determined by calculating relative risks and their 95% confidence intervals.

Advanced data analysis plan:

According to the suggestion from research review committee we will involve one health economist to conduct the cost analysis among two groups. The supportive variables will be inserted in the updated CRF.

Data collection procedure:

- Questionnaires will be visually scanned soon after interview and marked for omissions, inconsistencies or mistakes that will be addressed immediately.
- Data will be entered into a personal computer using Statistical Package for Social Sciences, version 15.0 Windows, (SPSS, Chicago, IL) after creating a template for each data entry file with appropriate logical and consistency checks.
- Data will be continuously entered as the data are being generated in hospital and laboratory.
- All data will be entered a second time, then consistency between two will be verified by matching with detected errors then necessary corrections will be made.
- In addition, data will be validated by a series of logical and range checks, producing summary statistics and tables.
- Data will be immediately copied on the hard disks of two computers as soon as data verification will be completed.

Intention to treat (ITT) analysis:

It is essential to mention that the children who will be "absconded" from our study after randomization due to any reason will also be included in our primary analysis and will be considered them as INTENTION TO TREAT (ITT) ANALYSIS. Thus, ITT analysis should be, any child who, after randomization fulfil any following criteria:

1. Criteria for treatment failure as defined in the proposal
2. Receives intubation and / or mechanical ventilation
3. Dies during hospitalization
4. Left against medical advice

Collection of Baseline Information

- All children within the defined age group suspected to have ALRI will be screened for study eligibility. Parents/ attending care givers of those fulfilling the eligibility, in application of the inclusion and the exclusion criteria, will be invited to provide their consent for enrollment of their children in the study. Upon signing a written informed consent, after providing information about the study and its interventions, possible benefits and risks, and voluntary nature of participation along with the right to withdraw children at any time after the initial consent without providing any reason, children will be enrolled. Case Record Form (CRF, ANNEXURE 1) will be used to collect relevant information such as medical history including nature and duration of illness, medication for current illness; socio-demographic characteristics such as sex, religion, parental age with education, parents occupation and smoking history, monthly family income, number of siblings, number of rooms, and cooking in bed room. Information will also be collected about child's feeding practice such as history of breast feeding, formula or other complementary feeding, and immunisation status; family history of tuberculosis, recent respiratory tract infection of any family members and past history of child's pneumonia, and history of exposure of the child to sun would be recorded. Examination findings to be recorded include pulse and respiratory rate, axillary temperature, anthropometric measurement, chest auscultation findings, chest wall in drawing, oxygen saturation, presence of cyanosis, and mental status (normal, irritable, lethargic). Weight of each child will be measured by electronic weighing scale with a precision of 0.1 kg and height/length will be measured using a locally made length board with precision of 0.1cm. Fever will be defined when the axillary temperature is 38℃ or greater. Respiratory rate will be counted for full 60 seconds by exposing the trunk when the child is awake and calm, and presence of lower chest wall in drawing will be noted at the same time. Respiratory rate will be counted for two times and if they differ by more than 5 breaths per minute then a third reading will be made, and the average of two closest respiratory rates (not deviating by 5 or greater number) will be regarded as the actual rate. Oxygen saturation will be measured using a Pulse Oxymeter (Nellcor Puritan Bennett Inc. N-560, Made in Korea) with a probe on a finger or toe when the child breathes in room air. Oxygen saturation of 90% or lesser will be defined as hypoxia which will be the indication for oxygen therapy. <u>If the child is sick we may require taking 5 mL of blood sample (approximately 1 tea spoon full) according to usual hospital management protocol.</u>

Management of diarrhea:

Children presented with diarrhea will be assessed for signs of dehydration (*Annex 2*). Dehydration will be corrected by oral rehydration saline unless there is specific indication of parenteral rehydration. The indications are: 1) Severe dehydration, 2) Paralytic ileus, 3) Persistent vomiting, 4) High purging, 5) Septic shock. All children >6 months old will be given Zinc supplementation for 10 days. Antibiotics are only recommended in dysentery, cholera or other microbiological confirmed cases.

Ethical Assurance for Protection of Human rights

Describe the justifications for conducting this research in human participants. If the study needs observations on sick individuals, provide sufficient reasons for using them. Indicate how participants' rights will be protected, and if there would be benefit or risk to each participants of the study. Discuss the ethical issues related to biomedical and social research for employing special procedures, such as invasive procedures in sick children, use of isotopes or any other hazardous materials, or social questionnaires relating to individual privacy. Discuss procedures safeguarding participants from injuries resulting from study procedures and/or interventions, whether physical, financial or social in nature. [Please see Guidelines]

The study will be performed in compliance with the 'Declaration of Helsinki' (2000), the International Conference of Harmonization (ICH), Tripartite Guidelines, Guideline for Good Clinical Practice (GCP). These procedures ensure the protection of the rights and the integrity of the study participants, adequate and correct conduct of all study procedures, adequate data collection, adequate documentation and adequate data verification.

The study will only be initiated after it has been approved by the Research Review Committee (RRC) and the Ethical Review Committee (ERC) of icddr,b. Before enrolment signed informed consent will be obtained from the adults and from the parents/guardians of the children. The consent form will be written in Bangla in a language and format that will be easily understood by the study subject of even little or no educational background. The consent form will be read out to the care-giver/legal guardian/parent of the study subject if he/she is unable to read. Signed consent or the left thumb impression will be obtained from the care-giver/legal guardian/parent for participation of the children in the study. Consent will be taken both for appendices 5a, b and c).

Data Safety Monitoring Plan (DSMP)

All clinical investigations (biomedical and behavioural intervention research protocols) should include the Data and Safety Monitoring Plan (DSMP) to provide the overall framework for the research protocol's data and safety monitoring. It is not necessary that the DSMP covers all possible aspects of each element. When designing an appropriate DSMP, the following should be kept in mind.

a) All investigations require monitoring;
b) The benefits of the investigation should outweigh the risks;
c) The monitoring plan should commensurate with risk; and
d) Monitoring should be with the size and complexity of the investigation.

Safety monitoring is defined as any process during clinical trials that involves the review of accumulated outcome data for groups of patients to determine if any treatment procedure practiced should be altered or not

---

Data safety Monitoring will be rigorously performed throughout the course of the study mainly by an independent DSMB who will visit randomly to review the forms for completeness, legibility, and internal consistency. icddr,b's ERC required reporting of all serious adverse events (SAE) within 24 hours; for this study all adverse events (AE) will also be reported by the study investigators to the DSMB within 24 hours of their occurrences using a standard case reporting form. As this is an open trial the implications of an adverse event can be better interpreted throughout the conduct of the trial than would be possible if any blinding was

involved. The DSMB will have full access to all case reporting forms and trial data, not only every 6 months, but whenever it is appropriate if there are any concerns over excess adverse events or unbalanced events between the three study groups. All aspects of intervention and interpretation will be performed according to rigorously standardized standards of procedures (SOPs).

Use of Animals

Describe if and the type and species of animals to be used in the study. Justify with reasons the use of particular animal species in the research and the compliance of the animal ethical guidelines for conducting the proposed procedures.

This study does not involve use of animals

Collaborative Arrangements

Describe if this study involves any scientific, administrative, fiscal, or programmatic arrangements with other national or international organizations or individuals. Indicate the nature and extent of collaboration and include a letter of agreement between the applicant or his/her organization and the collaborating organization.

Not applicable

Facilities Available

Describe the availability of physical facilities at site of conduction of the study. If applicable, describe the use of Biosafety Level 2 and/or 3 laboratory facilities. For clinical and laboratory-based studies, indicate the provision of hospital and other types of adequate patient care and laboratory support services. Identify the laboratory facilities and major equipment that will be required for the study. For field studies, describe the field area including its size, population, and means of communications plus field management plans specifying gender considerations for community and for research team members.

The International Centre for Diarrhoeal Disease Research, Bangladesh (icddr,b) has multi-disciplinary international and national scientific research staffs. Both the hospitals of icddr,b have adequate facilities for the management of acutely ill pediatric patients. Dhaka hospital of icddr,b additionally has a dedicated intensive care unit for management of seriously ill pediatric patients as well as a respiratory ward for admitting pneumonia patients. There is backup generator for continuous power supply. We have refrigerator (-20°C) and backup generator for 24 hours supply. According to the suggestion from the research review committee, we plan to store the blood sample for future molecular study, to identify the causative organism for developing serious infection.

Literature Cited

Identify all cited references to published literature in the text by number in parentheses. List all cited references sequentially as they appear in the text. For unpublished references, provide complete information in the text and do not include them in the list of Literature Cited. There is no page limit for this section, however, exercise judgment in assessing the "standard" length.

1. Rudan I, Boschi-Pinto C, Biloglav Z, et al. Epidemiology and etiology of childhood pneumonia. Bulletin of the World Health Organization 2008;**86**(5):408-16B
2. Harris M, Clark J, Coote N, et al. British Thoracic Society guidelines for the management of community acquired pneumonia in children: update 2011. Thorax 2011;**66**(Suppl 2):ii1-ii23
3. Margolis P, Gadomski A. Does this infant have pneumonia? Jama 1998;**279**(4):308-13
4. Wardlaw T, Salama P, Johansson EW, et al. Pneumonia: the leading killer of children. The Lancet 2006;**368**(9541):1048-50
5. Organization WH. Technical bases for the WHO recommendations on the management of pneumonia in children at first-level health facilities. 1991
6. Peterson S, Nsungwa-Sabiiti J, Were W, et al. Coping with paediatric referral—Ugandan parents' experience. The Lancet 2004;**363**(9425):1955-56
7. Addo-Yobo E, Chisaka N, Hassan M, et al. Oral amoxicillin versus injectable penicillin for severe pneumonia in children aged 3 to 59 months: a randomised multicentre equivalency study. The Lancet 2004;**364**(9440):1141-48
8. Hazir T, Fox LM, Nisar YB, et al. Ambulatory short-course high-dose oral amoxicillin for treatment of severe pneumonia in children: a randomised equivalency trial. The Lancet 2008;**371**(9606):49-56
9. Addo-Yobo E, Anh DD, El-Sayed HF, et al. Outpatient treatment of children with severe pneumonia with oral amoxicillin in four countries: the MASS study. Tropical Medicine & International Health 2011;**16**(8):995-1006
10. Bhutta ZA, Das JK, Walker N, et al. Interventions to address deaths from childhood pneumonia and diarrhoea equitably: what works and at what cost? The Lancet 2013;**381**(9875):1417-29
11. Walker CLF, Rudan I, Liu L, et al. Global burden of childhood pneumonia and diarrhoea. The Lancet 2013;**381**(9875):1405-16
12. File TM. Community-acquired pneumonia. The Lancet 2003;**362**(9400):1991-2001
13. Fonseca W, Hoppu K, Rey LC, et al. Comparing pharmacokinetics of amoxicillin given twice or three times per day to children older than 3 months with pneumonia. Antimicrobial agents and chemotherapy 2003;**47**(3):997-1001
14. Organization WH. Revised WHO classification and treatment of childhood pneumonia at health facilities–Evidence summaries, 2015.
15. Brogden R, Heel R, Speight T, et al. Amoxycillin injectable: a review of its antibacterial spectrum, pharmacokinetics and therapeutic use. Drugs 1979;**18**(3):169-84

Budget [Please add]

Donor : BMGF (Fund for capacity building)

Project Period (tentative): 12 months (Aug'17 to Jul'18)

(Amount in USD)

	**Effort %**	**Salary %**	**Unit Cost/ Month**	**Cost in USD**
**<u>Salaries and Benefits:</u>**				
Project Research Physician x 1 positions	100%	100%	560	7,112
Study Nurse x 4 position	100%	100%	350	17,780
Health Worker (GS1) x 2 positions	100%	100%	295	7,493
			**Sub Total**	**32,385**
**Travel:**				
IRB cost				1,400
Local travel				3,000
			**Sub Total**	**4,400**
**Supplies and Materials:**				
Stock and non stock (logistics items)				2,500
Drugs & Medical supplies				800
			**Sub Total**	**3,300**
**Indirect Cost (0%)**				**-**
			**Total Cost**	**40,085**

Budget Justifications

Please provide one page statement justifying the budgeted amount for each major item, including the use of human resources, major equipment, and laboratory services.

This study is only concerning with the incurring costs and aim to serve as an important reference in the service delivery in Dhaka Hospital. The major cost involved in our study is the salary support of the study personnel's. Another cost includes the direct and indirect costs. Details of the justification are given below:

**Major line items**	**Justification**
Human resource	*Project Research Physicians (PRP):* (100% FTE, 1 persons; US$ 7,112 salary) One physician will be assigned from 8:30-5 PM (6 days a week) for 12 months for screening and enrolment of the patient. Additionally, he/she will co-ordinate with all clinical activities including obtaining medical history, consents, performing clinical assessment, and prescribing treatment to enrolled patients.<br><br>*Study Nurse (SN):*) (100% FTE, 4 SN, US$ 17,780 salary): Four nurses will be recruited to cover the 24 hours duties in the whole study period. Nurses will be responsible for taking routine clinical care of study patients and monitor the vital signs on enrolment and 8 hourly.<br><br>*Health Workers* (HW) (100% FTE, 2 HW, US$ 7,493 salary): Two HW will be recruited for the whole study period. HWs will be responsible for subject selection and motivation. HWs will be responsible for collection, prompt transportation of sample from ward to laboratory (if required), escorting patient for radiology tests etc.
Travel	Total US$ 3,000 will be used for the transportation of study personnel's in weekends and in after office hour as per centre policy.
IRB approval	Total US$ 1,400 will be used for the essential charges for IRB approval according to centre's policy.
Supplies and Materials	Total US$ 3,300 will be used for purchase of emergency drug item and other supportive items such as: Stethoscope, Sphygmomanometer, stationary items, emergency drugs, printing cost, etc. One dedicated personal computer (PC) will be purchased for data entry purpose.

Other Support

Describe sources, amount, duration, and grant number of all other research funding currently granted to PI or under consideration.

Biography of the Investigators

Provide biographical data in the following format for all key personnel including the Principal Investigator. Copy the same format for each of them.
Note: Biography of the External Investigators may, however, be submitted in the format as convenient to them..

1 **Name: Dr. Lubaba Shahrin**

2 **Present Position:** Associate Scientist, NCSD & Lead, ARI ward, Dhaka Hospital

3 **Educational background:** (last degree and diploma & training relevant to the present research proposal)

	Institution	Year
Degree	FCPS, Bangladesh College of Physicians and Surgeons	2012
Degree	MBBS, University of Dhaka	2003
Training	Fellowship training in infectious disease from Weill Cornell Medical College Hospital, New York	2013
Training	Post graduate training in Pediatrics, Bangabandhu Sheikh Mujib Medical University (BSMMU)	2005-2006<br>2011-2012
Training	Post graduate training in Dhaka Medical College Hospital (Paediatrics)	2010

4 **Ethics Certification:**

		If Yes		
		Issuing Authority	Registration No	Valid Until
No ☐	Yes ☒	NIH	1932727	Issued 07/12/2015

Certificate of Completion

The National Institutes of Health (NIH) Office of Extramural Research certifies that **Lubaba Shahrin** successfully completed the NIH Web-based training course “Protecting Human Research Participants”.

Date of completion: 12/07/2015

Certification Number: 1932727

5 **List of ongoing research protocols/ activities**

Protocol/ Activity Number	Role in the protocol/ activity (PI, Co-PI, Co-I)	Starting date	End date	Percentage of time
PR-16051	Co-I	15.01.2016	14.01.2019	20%
PR-15113	Co-PI	01.10.2015	31.03.2017	12%

6 Publications

Types of publications	Numbers
a. Original scientific papers in peer-review journals	13
b. Peer reviewed articles and book chapters	1
c. Papers in conference proceedings	1
d. Letters, editorials, annotations, and abstracts in peer-reviewed journals	0
e. Working papers	2
f. Monographs	0

7. Five recent publications including publications relevant to the present research protocol

1. **Shahrin L**, Chisti MJ, Huq S, Nishath T, Christy MD, Hannan A, et al. Clinical Manifestations of Hyponatremia and Hypernatremia in Under-Five Diarrheal Children in a Diarrhea Hospital. Journal of tropical pediatrics. 2016:fmv100.
2. Chisti MJ, Salam MA, Ahmed T, Shahid ASMSB, Shahunja KM, Faruque ASG, **Shahrin L**, et al. Lack of BCG vaccination and other risk factors for bacteraemia in severely malnourished children with pneumonia. Epidemiology and Infection. 2015;143(4):799-803.
3. **Shahrin L**, Leung DT, Matin N, Pervez MM, Azim T, Bardhan PK, et al. Characteristics and Predictors of Death among Hospitalized HIV-Infected Patients in a Low HIV Prevalence Country: Bangladesh. Plos One. 2014;9(12)
4. **Shahrin L**, Leung DT, Matin N, Kawser CA, Pervez MM, Chisti MJ. Clinical profile of hospitalized HIV-infected children in Bangladesh, a low-HIV-prevalence country. Paediatrics and International Child Health. 2014;34(2):133-7.
5. **Shahrin L**, Chisti M, Huq S, Munirul Islam M, Golam Faruque A. Intractable Diarrhoea with Recurrent Hypernatremia: Experiences of Management Difficulties from a Diarrhoeal Treatment Centre of Bangladesh. J Clin Case Rep. 2014;4(460):2.

Biography of the Investigators

1. Name: Dr. Mohammod Jobayer Chisti

2 Present Position: Senior Scientist, NCSD, & Head, Clinical Research, Hospitals, & Clinical Lead, ICU & Consultant Physician, ARI ward, Dhaka Hospital, icddr,b; Dhaka, Bangladesh.

3 Educational background:

**Degree**	**Year**	**Institution**	**Comment**
PhD (Paediatric Respiratory Medicine)	June 2011- June 2014	Royal Children's Hospital, The University of Melbourne (UOM), Melbourne, Australia	Passed and received the nomination of the Chancellor`s Prize in the UOM
MMed (Master of Medicine) [Paediatrics]	March 2008 - March 2010	Royal Children's Hospital, The University of Melbourne, Melbourne, Australia	First Class Honours (H_1)
MBBS (Bachelor of Medicine and Bachelor of Surgery	January 1995	Sylhet MAG Osmani Medical College, Sylhet (Chittagong University)*	Pass

Ethics Certification:

		If Yes		
		Issuing Authority	Registration No	Valid Until
No ☐	Yes ☒	NIH	1933932	Issued 12/08/2015

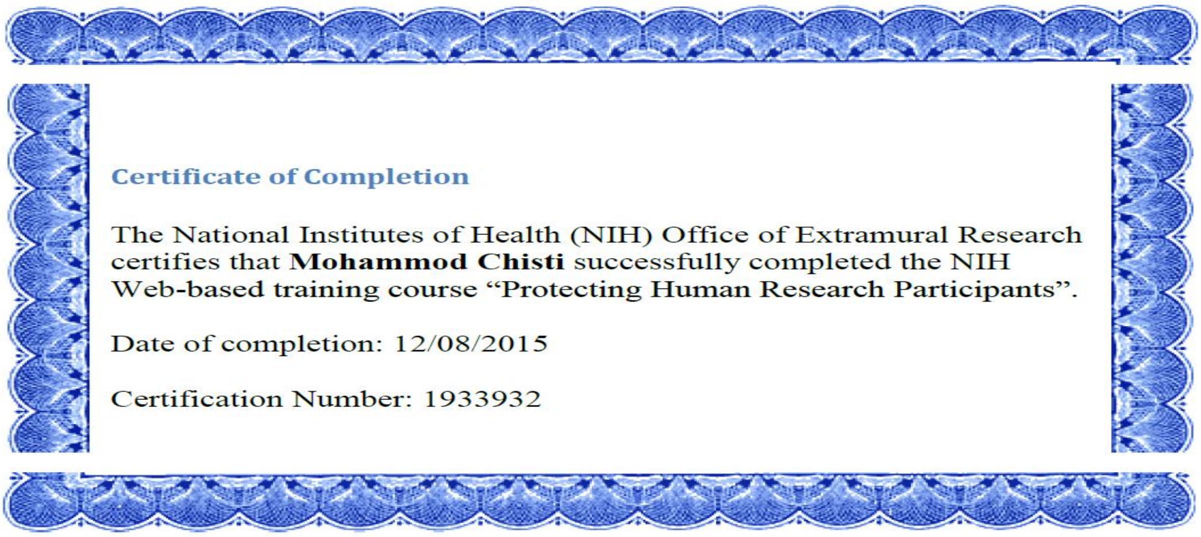

Certificate of Completion

The National Institutes of Health (NIH) Office of Extramural Research certifies that **Mohammod Chisti** successfully completed the NIH Web-based training course “Protecting Human Research Participants”.

Date of completion: 12/08/2015

Certification Number: 1933932

RBM certificate

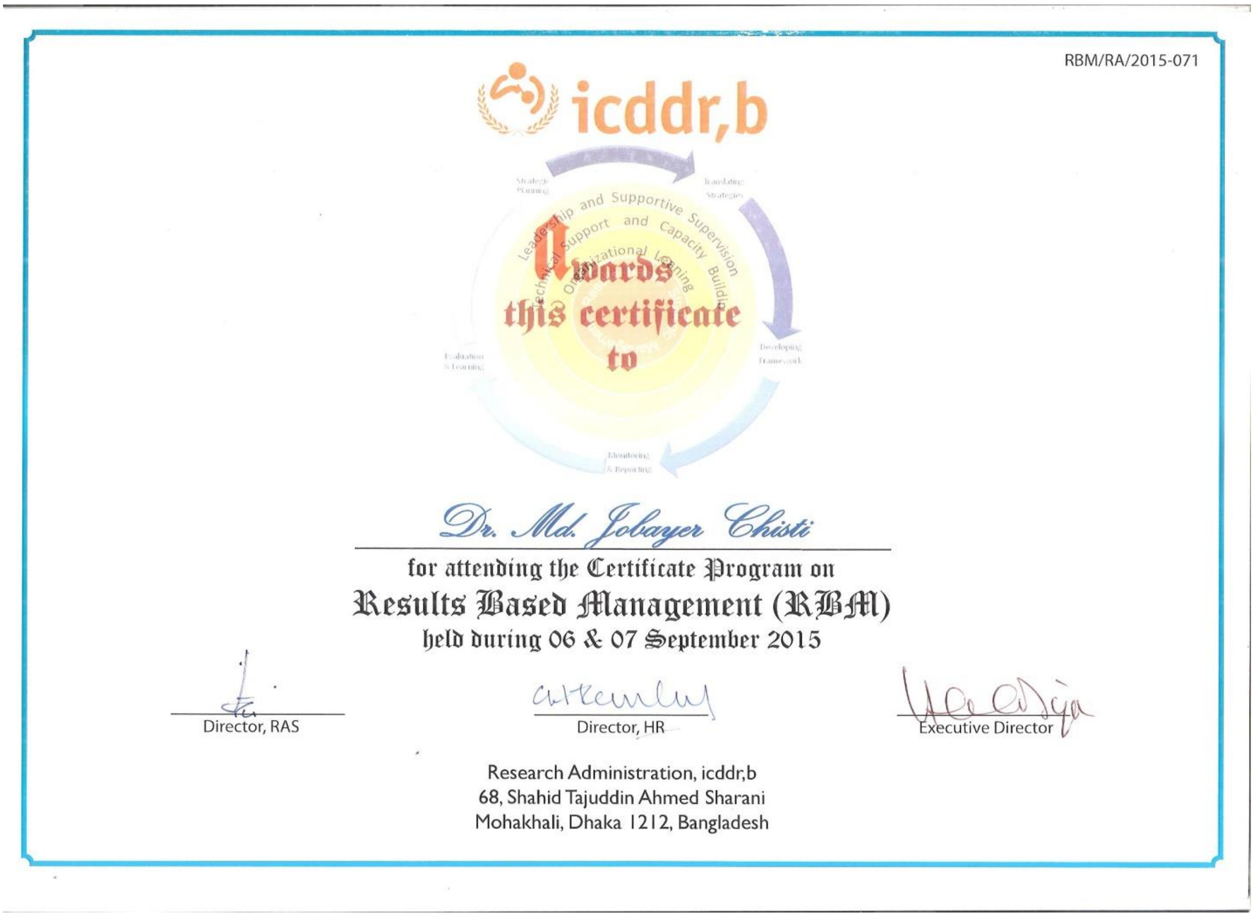

RBM/RA/2015-071

icddr,b

Awards this certificate to

Dr. Md. Jobayer Chisti

for attending the Certificate Program on

Results Based Management (RBM)

held during 06 & 07 September 2015

Director, RAS

Director, HR

Executive Director

Research Administration, icddr,b

68, Shahid Tajuddin Ahmed Sharani

Mohakhali, Dhaka 1212, Bangladesh

List of ongoing research protocols

Protocol/ Number	Role	Starting date	End date	Percentage of time
Antibiotics for Children with Severe Diarrhoea (ABCD) Trial/PR-16041	PI	01-04-2016	31-12-2019	50%
The Childhood Acute Illness & Nutrition Network: building the evidence base for care of acutely ill, undernourished children in limited resource settings: cohort study/ PR-16051	PI	01-07-2016	31-12-2018	20%
Tolerability and Acceptability of Fibersol-2 (Resistant Maltodextrin) in healthy and diarrheal children followed by a randomized clinical trial to evaluate the efficacy of Fibersol-2 in diarrheal children 1-3 years old/PR-16091	PI	23-04-2015	22-04-2016	20%
Non-respiratory biomarkers to diagnose and monitor response in pediatric tuberculosis (multi-centre study)/PR-15075	Co-PI	01-01-2016	31-12-2017	5%
Effectiveness trial of day-care versus usual care management of severe pneumonia with malnutrition in children using the existing health system of Bangladesh/PR-14066	Co-I	01-11-2015	31-10-2018	5%

Publications

Types of publications	Numbers
a. Original scientific papers in peer-review journals (as primary author, senior author and co-author)	139
b. Original scientific papers in peer-review journals (in press) (as primary author, senior author and co-author)	2
c. Peer reviewed book chapters (as primary author and co-author)	9
d. Papers in conference proceedings (as primary author and co-author)	66
e. Letters, editorials, annotations, and abstracts in peer-reviewed journals	6
f. Working papers (as a primary author, senior author)	6
g. Monographs	1

Link to PUBMED: http://www.ncbi.nlm.nih.gov/pubmed/?term=chisti+j+or+chisti+mj
Research gate: http://www.researchgate.net/profile/Mohammod_Chisti/publications

6 Five recent publications including publications relevant to the present research protocol

1. **Chisti MJ**, Shahid ASMSB, Shahunja KM, Bardhan PK, Faruque ASG, Shahrin L, Das SK, Barua DK, Hossain MI, Ahmed T. Ileus in children presenting with diarrhea and severe acute malnutrition: a chart review. *PLoS Neglected Tropical Diseases* 2017; 11: e0005603
2. **Chisti MJ**, Duke T, Salam MA, Shahunja KM, Shahid ASMSB, Bardhan PK, Faruque ASG, Ahmed T. Impact of diarrhea on the clinical presentation and outcome of severe pneumonia in Bangladeshi children. *The Pediatric Infectious Disease Journal* 2016; 35: 1161-1162

3. **Chisti MJ**, Salam MA, Smith JH, Ahmed T, Pietroni MAC, Shahunja KM, Shahid ASMSB, Faruque ASG, Ashraf H, Bardhan PK, Sharifuzzaman, Graham SM, Duke T. Bubble CPAP compared to standard- and high-flow oxygen therapy for children with severe pneumonia and hypoxaemia in Dhaka, Bangladesh: a randomized trial. *Lancet* 2015; 386 (9998): 1057-65
4. **Chisti MJ**, Graham SM, Duke T, Ahmed T, Ashraf H, Faruque ASG, La Vincente S, Banu S, Raqib R, Salam MA. A prospective study of the prevalence of tuberculosis and bacteraemia in Bangladeshi children with severe malnutrition and pneumonia including an evaluation of Xpert MTB/RIF assay. *PLoS ONE* 2014; 9: e93776
5. **Chisti MJ**, Salam MA, Ashraf H, Faruque ASG, Bardhan PK, Hossain MI, Shahid ASMSB, Shahunja KM, Das SK, Imran G, Ahmed T. Clinical risk factors of death from pneumonia in children with severe acute malnutrition in an urban critical care ward of Bangladesh. *PLoS ONE* 2013; 8: e73728

Biography of the Investigator:

1. **Name: Dr.Tahmeed Ahmed**

2. **Present Position:** Senior Director, Nutrition & Clinical Services Division, icddr,b and Professor of Public Health Nutrition, James P. Grant School of Public Health, BRAC University

3. **Educational background**:

Degree	Institution	Year
PhD	University of Tsukuba, Japan	1996
MBBS	University of Dhaka	1983
Training	Clinical training in Pediatrics, University of Tsukuba Hospital	1990-1992
Training	Residential training in Pediatrics, Dhaka Shishu Hospital	1989-1990

4. **Ethics Certification:**

		Issuing Authority	Registration No	Valid Until
No ☐	Yes ☒	NIH	1933611	Issued on 12 August 2015

5. **List of ongoing research protocols/ activities**

Protocol/ Activity Number	Role in the protocol/ activity (PI, Co-PI, Co-I)	Starting date	End date	Percentage of time
2008-020, MAL-ED	PI	Nov 2008	March 2017	40
PR-11005, CMAM	PI	June 2011	June 2017	30
Aflatoxin	Co-I	February 2013	May 2016	5
Hypernatremia follow up	Co-I	January 2016	January 2017	-
PR 15101, Emollient therapy for severe acute malnutrition	Co-PI	January 2016	March 2018	10
Bangladesh Environmental Enteric Dysfunction Study	PI	November 2015	November 2019	15

6. Publications

Types of publications	Numbers
a. Original scientific papers in peer-review journals	162
b. Peer reviewed articles and book chapters	22
c. Papers in conference proceedings	25
d. Letters, editorials, annotations, and abstracts in peer-reviewed journals	5
e. Working papers	10
f. Monographs	1

7. Five recent publications including publications relevant to the present research protocol

1. Dey N, Wagner VE, Blanton LV, Cheng J, Fontana L, Haque R, Ahmed T, Gordon JI. Regulators of gut motility revealed by a gnotobiotic model of diet-microbiome interactions related to travel. Cell 2015 Sep 24;163(1):95-107
2. Chisti MJ, Salam MA, Smith JH, Ahmed T, Pietroni MAC, Shahunja KM, Shahid ASMSB, Faruque ASG, Ashraf H, Bardhan PK, Sharifuzzaman, Graham SM, Duke T. Bubble CPAP compared to standard- and high-flow oxygen therapy for children with severe pneumonia and hypoxaemia in Dhaka, Bangladesh: a randomized trial. *Lancet* 2015; 386 (9998): 1057-65
3. Platts-Mills JA, Babji S, Bodhidatta L, Gratz J, Haque R, Havt A, McCormick BJJ, McGrath M, Olortegui MP, Samie A, Shakoor S, Mondal D, Lima IFN, Hariraju D, Rayamajhi BB, Qureshi S, Kabir F, Yori PP, Mufamadi B, Amour C, Carreon JD, Richard SA, Lang D, Bessong P, Mduma E, Ahmed T, Lima AAAM, Mason CJ, Zaidi AKM, Bhutta ZA, Kosek M, Guerrant RL, Gottlieb M,Miller M, Kang G, Houpt ER, and The MAL-ED Network Investigators. Pathogen-specific burdens of community diarrhoea in developing countries: a multisite birth cohort study (MAL-ED). Lancet Glob Health 2015 Jul 20. [Epub ahead of print]
4. Christian P, Shaikh S, Shamim AA, Mehra S, Wu L, Mitra M, Ali H, Merrill RD, Choudhury N, Parveen M, Fuli RD, Hossain MI, Islam MM, Klemm R, Schulze K, Labrique A, de Pee S, Ahmed T, West KP, Jr. Effect of fortified complementary food supplementation on child growth in rural Bangladesh: a cluster-randomized trial. Int J Epidemiol 2015 Aug 14.
5. Ahmed T, Choudhury N, Hossain I, Tangsuphoom N, Islam MM, de Pee S, Steiger G, Fuli R, Sarker SA, Parveen M, West KP, Christian P. Development and acceptability testing of ready-to-use supplementary food made from locally available food ingredients in Bangladesh. BMC Pediatr 2014 Jun 27;14:164.

Certificate of Completion

The National Institutes of Health (NIH) Office of Extramural Research certifies that **Tahmeed Ahmed** successfully completed the NIH Web-based training course “Protecting Human Research Participants”.

Date of completion: 12/08/2015

Certification Number: 1933611

Biography of the Investigator:

1. **Name:** Dr. Sayeeda Huq

2. **Present Position:** Associate Scientist, Consultant Physician, Nutrition Ward, Nutrition & Clinical Services Division, icddr,b

3. **Educational background:**
(last degree and diploma & training relevant to the present research proposal)

1989-1996 MBBS (Bachelor of Medicine and Bachelor of Surgery), Bangladesh Medical College, Dhaka, Bangladesh.

2007 Masters of International public health, Department of Public Health, University of Sydney, Australia

4. Ethics Certification:

		If Yes		
		Issuing Authority	Registration No	Valid Until
**No** ☐	Yes ☒	NIH	1932845	Issued 12/07/2015

5. List of ongoing research protocols/ activities

Protocol/ Activity Number	Role in the protocol/ activity (PI, Co-PI, Co-I)	Starting date	End date	Percentage of time
PR#15113	PI	25-03-16	31-05-17	6%
PR# 09023	PI	1-10-09	30-06-13	25%
PR#09038	Co-I	01-08-12	31-12-14	10%
PR #10039	Co-I	20-01-12	19-01-15	5%
PR#11005	Co-I	18-08-11	31-12-15	5%
PR#12082	Co-I	1-08-13	30-11-15	25%

6.. Publications

Types of publications	Numbers
Original scientific papers in peer-review journals	25
Peer reviewed articles and book chapters	2
Papers in conference proceedings	11
Letters, editorials, annotations, and abstracts in peer-reviewed journals	3
Working papers	
Monographs	1

7. Five recent publications including publications relevant to the present research protocol

7.1**.** **S Huq,** , M I Hossain, MA Malek, ASG Faruque and M A Salam. Hypoglycaemia in under five children with diarrhoea. Journal of Tropical Pediatrics; 2007 Jun;53(3):197-201.

7.2. Sathish Subramanian, **Sayeeda Huq**,Tanya Yatsunenko,Rashidul Haque,Mustafa Mahfuz, Mohammed A.Alam,Amber Benezra,Joseph DeStefano,Martin F. Meier,Brian D. Muegge,Michael J. Barratt,Laura G. VanArendonk, Qunyuan Zhang, Michael A. Province,William A. Petri Jr,Tahmeed Ahmed,Jeffrey I. Gordon.

Persistent gut microbiota immaturity in malnourished Bangladeshi children. Nature http://dx.doi.org/10.1038/nature13421 (2014).

7.3. **S Huq**, Mark A.C. Pietroni, Hafizur Rahman, Mohammad Tariqul MA. Hereditary Spherocytosis. Journal of Health, Population and Nutrition , 2010 Feb; 28(1), 107-109.

7.4. Ahmed T, Islam M, Choudhury N, Hossain I, **Huq S**, Mahfuz M, Sarker SA.Results with complementary food using local food ingredients.Nestle Nutr Inst Workshop Ser. 2017;87:103-113. doi: 10.1159/000448960.2017 Mar 17.

7.5.Sumon Das, Jobayer Chisti, **Sayeeda Huq**, Mohammad Abdul Malek, Lana Vanderlee, Mohammed Abdus Salam, Tahmeed Ahmed, Abu Syed Golam Faruque, Abdullah Al Mamun. Changing trend of overweight and obesity and their associated factors in urban population of Bangladesh. Food and Nutrition Sciences, 2013,4,678-689.

Certificate of Completion

The National Institutes of Health (NIH) Office of Extramural Research certifies that **Sayeeda Huq** successfully completed the NIH Web-based training course "Protecting Human Research Participants".

Date of completion: 12/07/2015

Certification Number: 1932845

1. **Name: Dr. Farzana Afroze**

2. **Present Position:** Senior Medical Officer/Assistant Scientist, NCSD, icddr,b
3. **Educational background**: (last degree and diploma & training relevant to the present research proposal)

	Institution	Year
FCPS (Paediatrics)	Bangladesh College of Physicians and Surgeons (BCPS)	Jan, 2015
MBBS	Chittagong University	Jan, 2001
Post graduate training (Paediatrics)	BSMMU	2009-2010, 2014
Post graduate training (Paediatrics)	Dhaka Medical College Hospital	2007-2009

4. **Ethics Certification:**

If Yes

		Issuing Authority	Registration No	Valid Until
**No** ☐	Yes ☒	NIH	**2265620**	

Note: If the response is "no", please get certification from CITI or NIH before study initiation and submit a copy to the Committee Coordination Secretariat

5. List of ongoing research protocols/ activities

Protocol/ Activity Number	Role in the protocol/ activity (PI, Co-PI, Co-I)	Starting date	End	Percentage of time
Vasoactive Intestinal Peptide (VIP) in the pathogenesis of cholera- a descriptive study [PR-17008] (In review process)	PI			

6. Publications

SEE ATTACHED C.V.

Types of publications	Numbers
g. Original scientific papers in peer-review journals	6
h. Peer reviewed articles and book chapters	1
i. Papers in conference proceedings	
j. Letters, editorials, annotations, and abstracts in peer-reviewed journals	
k. Working papers	1
l. Monographs	

7. Five recent publications including publications relevant to the present research protocol

7.1 Afroze F, Ahmed T, Sarmin M, Smsb Shahid A, Shahunja KM, Shahrin L, Chisti MJ. Risk factors and outcome of Shigella encephalopathy in Bangladeshi children. PLoS Negl Trop Dis. 2017 Apr 28;11(4):e0005561. doi: 10.1371/journal.pntd.0005561. eCollection 2017 Apr.

7.2 Das R, Ahmed T, Saha H, Shahrin L, **Afroze F**, Shahid ASMSB, , K M Shahunja, Bardhan P.K, Chisti M.J et al; Clinical risk factors, bacterial aetiology, and outcome of urinary tract infection in children hospitalized with diarrhoea in Bangladesh, Epidemiology & Infection 2016: 1-7

7.3 Das SK, **Afroze F**, Ahmed T, Faruque ASG, Sarker SA, Huq S, Islam MM, Shahrin L, Matin FB, Mohammod J Chisti; Extreme hypernatremic dehydration due to potential sodium intoxication: consequences and management for an infant with diarrhea at an urban intensive care unit in Bangladesh: a case report, Journal of Medical Case Reports 2015, **9**:124

7.4 Sumon K Das, Mohammod J Chisti, Mokibul H Afrad, Mohammad A Malek, Shahnawaz Ahmed, Farzana Ferdous, Fahmida D Farzana, K M Shahunja, **Afroze F**, Mohammed A Salam, Tahmeed Ahmed, Abu SG Faruque, Jui Das, Peter John Baker and Abdullah Al Mamun; Gastroenteritis due to typhoidal Salmonella: a decade of observation at an urban and a rural diarrheal disease hospital in Bangladesh, BMC Infectious Diseases 2014, 14 :435 http://www.biomedcentral.com/1471-2334/14/435

7.5 Afroze F, Ahmed T, Sarker SA, Faruque ASG, Ashras H, Mohammod J Chisti et al; Predictors of Meningitis in Under Fifteen Children Attending an Intensive Care Unit of an Urban Large Diarrheal Disease Hospital in Bangladesh, Food and Nutrition Sciences 2014, 5: 169-17

Certificate of Completion

The National Institutes of Health (NIH) Office of Extramural Research certifies that **Farzana Afroze** successfully completed the NIH Web-based training course "Protecting Human Research Participants".

Date of completion: 01/09/2017.

Certification Number: 2265620

Biography of the Investigators

1 **Name:** Dr. Abu Sadat Mohammad Sayeem Bin Shahid
2 **Present Position:** Assistant Scientist, NCSD, icddr,b
3 **Educational background:** (last degree and diploma & training relevant to the present research proposal)

Degree	Institution	Year
MBBS	Shahjalal University of Science & Technology	2006

4 **Ethics Certification:**

		If Yes		
		Issuing Authority	Registration No	Valid Until
No ☐	Yes ☒	NIH	1636072	Issued 12/15/2014

Certificate of Completion

The National Institutes of Health (NIH) Office of Extramural Research certifies that **ABU SADAT BIN SHAHID** successfully completed the NIH Web-based training course “Protecting Human Research Participants”.

Date of completion: 12/15/2014

Certification Number: 1636072

5 **List of ongoing research protocols/ activities**

Protocol/ Activity Number	Role in the protocol/ activity (PI, Co-PI, Co-I)	Starting date	End date	Percentage of time
PR-16051	Co-I	10.01.2016	09.30.2019	100%

6 Publications

Types of publications	Numbers
g. Original scientific papers in peer-review journals	25
h. Peer reviewed articles and book chapters	0
i. Papers in conference proceedings	2
j. Letters, editorials, annotations, and abstracts in peer-reviewed journals	0
k. Working papers	1
l. Monographs	0

8. Five recent publications including publications relevant to the present research protocol

1. Chisti MJ, **Shahid ASMSB**, Shahunja KM, Bardhan PK, Faruque ASG, Shahrin L, Das SK, Barua DK, Hossain MI, Ahmed T. Ileus in children presenting with diarrhea and severe acute malnutrition: a chart review. *PLoS Neglected Tropical Diseases* 2017; 11: e0005603

2. Alam T, Ahmed T, Sarmin M, Shahrin L, Afroze F, Sharifuzzaman, Akhter S, Shahunja KM, **Shahid ASMSB**, Bardhan PK, Chisti MJ. Risk Factors for Death in Bangladeshi Children Under 5 Years of Age Hospitalized for Diarrhea and Severe Respiratory Distress in an Urban Critical Care Ward. Glob Pediatr Health.2017 Mar 30;4:2333794X17696685. doi: 10.1177/2333794X17696685. eCollection 2017.
3. Chisti MJ, Duke T, Salam MA, Shahunja KM, **Shahid ASMSB**, Bardhan PK, Faruque ASG, Ahmed T. Impact of diarrhea on the clinical presentation and outcome of severe pneumonia in Bangladeshi children. *The Pediatric Infectious Disease Journal* 2016; 35: 1161-1162

4. Chisti MJ, Salam MA, Smith JH, Ahmed T, Pietroni MAC, Shahunja KM, **Shahid ASMSB**, Faruque ASG, Ashraf H, Bardhan PK, Sharifuzzaman, Graham SM, Duke T. Bubble CPAP compared to standard- and high-flow oxygen therapy for children with severe pneumonia and hypoxaemia in Dhaka, Bangladesh: a randomized trial. *Lancet* 2015; 386 (9998): 1057-65

5. Chisti MJ, Salam MA, Ashraf H, Faruque ASG, Bardhan PK, Hossain MI, **Shahid ASMSB**, Shahunja KM, Das SK, Imran G, Ahmed T. Clinical risk factors of death from pneumonia in children with severe acute malnutrition in an urban critical care ward of Bangladesh. *PLoS ONE* 2013; 8: e73728

International Centre for Diarrhoeal Disease Research, Bangladesh (icddr,b)
Voluntary Consent Form

Protocol No. PR-17061 Version No. 1.2 Date: _ _ _ _ _ _ _ _

Protocol Title: Efficacy of two doses of parenteral Amoxicillin plus single dose Gentamicin compared to four doses of parenteral Ampicillin plus single dose Gentamicin in managing children hospitalized with WHO classified severe pneumonia: an open labelled randomized controlled clinical trial
Investigator's Name: Dr Lubaba Shahrin
Organization: icddr,b

Please feel free to ask any questions you may have about this study and the information given below. You will be given opportunity to ask questions will be answered. If you agree to our proposal of inclusion of your child in our study, we would provide you with a copy of this consent form.

Purpose of the research

Severe pneumonia is a serious infection in children which requires timely intervention. Management of severe pneumonia is done by appropriate antibiotics and supportive care. WHO recommendation is to use injection ampicillin four times a day for 3-5 days and injection gentamicin once a day for 3-5 days in children with severe pneumonia. In our study we want to compare the response of injection ampicillin with another similar and equally effective antibiotic (amoxicillin). The reason of choosing amoxicillin is that the dose is two times a day and it is cost effective and reduces chance of nosocomial infection. The response of the drugs will be compared by time difference of clinical improvement of the patients or time difference of disappearance of the sign and symptoms on admission. We are interested to compare the recovery time from the illness as well as treatment failure in both group. In that way we will be able to develop a better treatment for pneumonia in children.

Why invited to participate in the study?

As we are comparing the efficacy of two treatment regimen in the children with severe pneumonia, we have to enroll hospitalized patient. Your child has been identified because the child is admitted to hospital with severe pneumonia. We are doing this research to help to identify better ways to care for children in the future.

Methods and procedures [What is expected from the participants of the research study?]

- Your child will continue to receive usual care and treatment for the condition with which he/she is admitted
- We will review the medical records from your child's hospitalization, previous hospitalizations and hospitalizations that occur during study follow-up.
- We will perform detailed measurements of your child's nutritional status and food intake.
- If require we may perform a chest x ray to your child, which is non invasive and with minimum hazard.
- If your child is sick we may require taking small blood sample according to usual hospital management protocol.
- After the study has finished we may wish to contact you again, to ask if you are free of illness after discharge or not.

Risk and benefits

Some pain and discomfort will be experienced from the needle prick when blood samples are obtained. Very occasionally, taking blood samples might cause infection. Careful attention will however be paid to ensure that the sample is taken in a professional manner to avoid these problems. The benefits for your child taking part in this study will be extensive monitoring during whole hospitalization period, free of cost treatment, other logistic support and free medical consultation even after discharge from the study. The results of this study will be beneficial to society in the future as they may help improve care for children with malnutrition.

Privacy, anonymity and confidentiality

We shall assure that the privacy, anonymity and confidentiality of data/information identifying your patient will be strictly maintained. We would keep all the medical information, description of treatment, and results of laboratory tests performed on your patient confidential, and stored in a safe place, under lock and key under the

responsibility of the principal investigator. None other than the investigators of this research study; possible study monitor; regulatory authorities, such as the Ethical Review Committee (ERC) of icddr,b; and any law-enforcing agency in the event of necessity would have an access to the information. We want to inform you that data related to the study will not be sent outside the country for analysis. The name or identity of your child would not be disclosed while publishing the results of this study.

Future use of information

In case of future use of the information collected from the study, the investigators will arrange to provide that in such cases anonymous or abstracted information and data may be supplied to other researchers, which should not conflict with or violate the maintenance of privacy, anonymity and confidentiality of information identifying participants in any way.

Right not to participate and withdraw

Participation in research is voluntary. You are free to decide if you want your child to take part in research or not. Your child will still receive the recommended standard care whether or not you agree to take part. If you have decided to participate, you can change your mind any time and withdraw your child from the study. This will not affect your child's care now or in the future.

WHAT WILL HAPPEN TO THE SAMPLES?

The chest x ray will be preserved for study purpose upto 5 years after completion of the study. Blood will be stored for any future molecular analysis to identify the causative organism.

WHO WILL BE INVOLVED IN ACCESSING MY CHILD'S INFORMATION IN THIS STUDY?

All information on participants collected in this study will be stored in a confidential manner in locked, secured cabinets and password protected computers and will only be accessible to authorised study personnel. Data will be stored to the end of the study and analysis. Any report or publications on this study will not use participant's names or identities.

WHO HAS AUTHORIZED THIS RESEARCH TO TAKE PLACE?

The RRC and ERC of icddr,b have checked and approved this work.

WHAT IF I HAVE ANY QUESTIONS?

You can ask any of our workers anytime. You can also communicate with those involved in taking care of your child and this study. At any point of time during hospitalization and even after discharge you can contact with the PI (Dr Lubaba Shahrin) for any health related query of your child.

Principle of compensation

For any study related injuries, we will provide treatment in the hospital at free of cost.

If you agree to our proposal of enrolling you/your patient in our study, please indicate that by putting your signature or your left thumb impression at the specified space below

Thank you for your cooperation

____________________________________ ____________________

Signature or left thumb impression of Parent/ Guardian/ Attendant — Date

____________________________________ ____________________

Signature or left thumb impression of the witness — Date

____________________________________ ____________________

Signature of the PI or his/her representative — Date

(NOTE: In case of representative of the PI, she/he shall put her/his full name and designation and then sign)
(Name and contact phone of IRB Secretariat, RA, M. A. Salam Khan, Phone No: 9886498 or PABX 8860523-32 Extension. 3206).

Principle Investigator:

Dr Lubaba Shahrin, Associate Scientist, Head, ARI ward, Dhaka Hospital +88029827001-10 Ext 3391.
+88 01754598328

IRB Secretariat, RA, M. A. Salam Khan, Phone No: 9827084 or PABX 9827001-10, Extension. 3206;

Detailed Budget for the study titled:

Efficacy of two doses of parenteral Amoxicillin plus single dose Gentamicin compared to four doses of parenteral Ampicillin plus single dose Gentamicin in managing children under five with severe pneumonia: an open labelled randomized controlled clinical trial

Name of Principal Investigator: Dr Lubaba Shahrin

Protocol Number: PR-17061

Division: NCSD

BGT-288/2017

Title of Protocol: Efficacy of two doses of parenteral Amoxicillin plus single dose Gentamicin compared to four doses of parenteral Ampicillin plus single dose Gentamicin in managing children 3-59 months of age hospitalized with severe pneumonia: an open labeled randomized controlled clinical trial

Donor : BMGF (Fund for capacity building)

Project Period (tentative): 12 months (Aug'17 to Jul'18)

(Amount in USD)

	Effort %	Salary %	Unit Cost/ Month	Cost in USD
**Salaries and Benefits:**				
Project Research Physician	100%	100%	560	7,112
Study Nurse x 4 position	100%	100%	350	17,780
Health Worker (GS1) x 2 positions	100%	100%	295	7,493
			**Sub Total**	**32,385**
**Travel:**				
Local travel				3,000
			Sub Total	3,000
**Supplies and Materials:**				
Stock and non stock (logistics items)				2,500
Drugs & Medical supplies				800
			**Sub Total**	**3,300**
IRB Cost				**1,400**
			**Total Direct Cost**	**40,085**
**Indirect Cost (0%)**				-
			**Total Cost**	**40,085**

OH Calculation:

BuiltIn: IRB Cost	1,400
Direct Cost	38,685
OH Rate	4%

Lubaba 07/06/17

22/6/17

22/06/17

07/06/17

Budget Justification Form

BGT-288/2017

Project Title :Efficacy of two doses of parenteral Amoxicillin plus single dose Gentamicin compared to four doses of parenteral Ampicillin plus single dose Gentamicin in managing children hospitalized with WHO classified severe pneumonia: an open labelled randomized controlled clinical trial

Grant : ☒ New ☐ Existing -GR/BC Number:

Donor Name : BMGF

Budget Period : August' 17 to July'18

PI Name : Dr Lubaba Shahrin

PI's Email & Extn No : lubabashahrin@icddrb.org; Ext 3391

Division : NCSD

Budgted Amount : 40,085 USD

Approving Officer	Justification/Comments	Signature
Principle Investigator/ (PI/FPI)	PI will be responsible for overall activities of the project starting from project staff recruitment to reporting of the project activities.	Lubaba
Finance Manager/ Assigned Officer	Overhead 0%	[illegible]
Senior Budget Coordinator	Budget considers accurate:<br>☐ Project Period<br>☐ Employee CTC<br>☐ Employee FTE / Time Allocation<br>☐ Previous Actual (revision)<br>☐ Budget Narrative<br>☐ Rent<br>☐ HVAC<br>☐ Notes (if any)<br>☐ Inflation Adjustment<br>☐ Other Operating Expenses<br>☐ Capex<br>☐ FX Rate<br>☐ Activity/Protocol<br>☐ Audit Fee<br>☐ Overhead Rate ......<br>☐ Built-in OH	
Senior Manager, B&P	Considering the objective of the project such as. & Hospital service improvement & Cost reduction Budget may be approved @ 07.	[illegible] 22/6/17
Controller, Finance		
Director, Finance		Sign [illegible]<br>Date 22/6/17<br>Thomas Liam Barry

icddr,b

Check-List

Check-list for Submission of Research Protocol
For Consideration of the Research Review Committee (RRC)
[Please check all appropriate boxes]

1. Has the proposal been reviewed, discussed and cleared by all listed investigators?

   ☒ Yes ☐ No

   If the response is No, please clarify the reasons:

2. Has the proposal been peer-reviewed externally?

   ☒ Yes ☐ No ☐ External Review Exempted

   If the response is 'No' or "External Review Exempted", please explain the reasons:

   If the response is "Yes", please indicate if all of their comments have been addressed?

   ☒ Yes (please attach)

   ☐ No (please indicate reason(s)):

3. Has the budget been reviewed and approved by icddr,b's Finance?

   ☒ Yes ☐ No (reason):

   ____

4. Has the Ethics Certificate(s) been attached with the Protocol?

   ☒ Yes ☐ No

   If the answer is 'No', please explain the reasons:

____
Signature of the Principal Investigator Date___________

Guidelines for Preparing Abstract for ERC

The Ethical Review Committee will not consider any application that does not include an abstract summary. The abstract should summarise the purpose of the study, the methods and procedures to be used, by addressing each of the following items. If an item is not applicable, please note accordingly, describing the reason:

1. Describe the requirements for a 'study population' and explain the rationale for inclusion of special groups in this study population, such as children or groups whose ability to give voluntary informed consents might be compromised.

2. Assess and describe potential risk(s) – physical, psychological, social, legal or other, and also assess their likelihood and seriousness. If research methods are anticipated to involve potential risks, describe alternate methods, if any, which were considered and why they will not be used.

3. Describe procedures for protecting against or minimising potential risks, and an assessment of their likely effectiveness.

4. Include a description of the methods for safeguarding confidentiality and protecting anonymity.

5. When there are potential risks to the participants, or when the privacy of the individual may be affected, the investigators are required to obtain a written informed consent, duly signed by the prospective participants. For minors and individuals with compromised ability to provide a valid consent, informed consent must be obtained from their parents or legal guardians. Describe consent procedures to be followed including how and where informed consent will be obtained.

    a) If signed consent will not be obtained, explain why this requirement should be waived and provide an alternative procedure that would be used.

    b) If information is to be withheld from a participant, provide justification for this course of action.

    c) If there is a potential risk to the participant or privacy of the individual might be affected while applying any particular procedure include a statement in the consent form to clarify whether or not compensation and/or treatment will be available and who will support the costs.

6. If study involves an interview, describe the place and processes, and approximate length of the interview.

7. Assess the potential benefits to be gained or risk the individual participants might be subjected to, and also the benefits that might accrue to the society in general as a result of the planned work. Clarify if and how the benefits outweigh the risks.

8. State if the activity requires the use of records (hospital, medical, birth, death or other), organs, tissues, body fluids, the foetus or the abortus.

    The statement to the potential participants should include information specified in item 2,3,4,5(c) and 7, and also indicate the approximate time they would be required to remain in the activity.

1Annexure:

Annex 1:

WHO algorithm (2013) for the classification of the severity of pneumonia (children presenting with cough and/or respiratory difficulty)

Age	Clinical parameter	Diagnosis
2 months – 59 months of age	Cough or difficulty in breathing with:<br>▪ Oxygen saturation <90% or central cyanosis<br>▪ Severe respiratory distress (e.g. grunting, very severe chest in drawing)<br>▪ Signs of pneumonia with a general danger sign (inability to breastfeed or drink, lethargy or reduced level of consciousness, convulsion)	Severe pneumonia
	▪ Fast breathing:<br>≥ 50 breaths/min in a child aged 2-11 months<br>≥ 40 breaths/min in a child aged 1-5 years<br>▪ Chest in drawing	Pneumonia
	▪ No signs of pneumonia or severe pneumonia	No pneumonia: cough or cold

Children with any form of WHO-defined pneumonia with **severe clinical malnutrition (SAM)** were also considered to have severe pneumonia. Diagnosis of pneumonia should be confirmed after full correction of dehydration or in absence of dehydration.

Annex 2:

WHO algorithm for the classification of the signs of dehydration

**Assess**	Condition	Normal	Irritable/Less active*	Lethargic/comatose
	Eyes	Normal	Sunken	-
	Tongue	Normal	Dry	-
	Thirst	Normal	Thirsty (drinks eagerly)	Unable to drink*
	Skin pinch	Normal	Goes back slowly*	-
	Radial pulse	Normal	Low*	Uncountable or absent*
**Diagnosis**		No sign of dehydration	If at least 2 signs, including one of the *- marked signs, are present, diagnose Some dehydration	If some dehydration + one of the *- marked signs are present, diagnose Severe Dehydration
**Treatment**		•Prevent dehydration with ORS<br>•Reassess periodically	•Rehydrate with ORS<br>•Frequent reassessment	•Rehydrate with IV fluids and ORS<br>•More frequent reassessment

Annex 3: Classification of severe acute malnutrition (SAM) in children aged 6-59 months

- Mid-upper arm circumference <115 mm
- Weight-for-height Z-score (WHZ) <-3 (annex 1 and annex 2)
- Bilateral pedal edema (kwashiorkor and marasmic kwashiorkor are 'edematous malnutrition')

**Steps of Acute Phase management of SAM**	
**Step 1**	Treat/prevent hypoglycemia
**Step 2**	Treat/prevent hypothermia
**Step 3**	Treat/prevent dehydration
**Step 4**	Correct electrolyte imbalance
**Step 5**	Treat/prevent infection
**Step 6**	Correct micronutrient deficiencies
**Step 7**	Start cautious feeding

Annex 4 : Case Record Form (CRF)

Efficacy of two doses of parenteral Amoxicillin plus single dose Gentamicin compared to four doses of parenteral Ampicillin plus single dose Gentamicin in managing children hospitalized with WHO classified severe pneumonia: an open labelled randomized controlled clinical trial

1. Study Number: _________

2. Date of admission: (Day/Month/Year) _____/_____/_________

3. Date of Enrollment: (Day/Month/Year) _____/_____/_________

4. Date of Discharge / transfer to NRU (Day/Month/Year) _____/_____/_________

5. Hospital ID number: /__/__/__/__/__/__/__/

6. Age: ____ months ____days

7. Sex: 1=Male 2= Female ☐

Please check the following on admission:

8. **Is the child between 3 - 59 months of age? 1=Yes, 2=No** ☐

9. Does the child fit the clinical WHO criteria for severe pneumonia? 1=Yes, 2=No ☐

10. Has the mother/guardian given consent to participate in this study? 1=Yes, 2=No ☐

(***Question 8 to 10, If Answer is 'NO' in any one, then the child should not be included in the study)***

For those that satisfy study inclusion criteria please proceed with enrollment.

11. Study group: (1) Intervention group, (2) Control group ☐

Socio-demographic information

12. Current residence: 1. Village 2.Township ☐

SECTION 1: BACKGROUND INFORMATION

13. Date of birth of the patient: (dd/mm/yy) (if known)
___/___/_____

14. Birth weight of the patient in kg (if known):
____________ kg

15. Place of normal residence of the patient
1=within Dhaka city, 2=within Dhaka district, 3= outside Dhaka district ☐

16. Gestational age at birth (weeks) [code 88, if not known) ☐☐

17. H/O perinatal asphyxia?

1=yes, 2=no, 3=don't know

18. Mother's occupation

1=House wife, 2= Garments worker, 3= Day labourer, 4= private job,
5= govt.job, 6= others, 7=Maid servant, 8=Street begger,9= Not Applicable

19. Father's occupation
1=Unemployed, 2= Day labourer, 3=Hawker, 4=Garments worker,
5= Private job, 6=Govt.job, 7=other, 8= Rickshaw puller,9=Not Applicable

20. Type of locality where care giver live?

1=Slum area , 2=Common housing area, 3= Residential area, 4= Village, 5= others

21. What was the first symptom of the patient?
1=Cold/Runny nose, 2=Cough, 3=Fever, 4=Diarrhoea, 5=Others 6=Respiratory distress, 7= Vomiting, 8=Convulsion ,

22. Whom did you consult first for this episode of illness of your child ?

1= Qualified doctor, 2= Homeopath doctor or spiritual adviser
3= Quack, 4= Senior family member, 5= Traditional healer,6=Others ,7= None

23. Why did delay occur during hospitalization? (if any)
1= Financial constraint, 2= To take care of home, 3= Under treatment of quack

4= Under homeopath or spiritual treatment, 5= Could not realise the problem
6= Single at home, 7= others (please specify), 9=N/A

24. Socioeconomic status (family):
1=Rich (>20000 taka/m), 2=Middle class (10000-20000 taka/m)
3=Poor (income <10000taka/m)

25. Intake of Vit-A capsule within last 6 months
1=yes, 2=no,3=don't know

26. History of measles within 6 months prior to admission
1= never, 2= within last 3 months, 3= within 3-6 months or more,4=don't know

27. Method of feeding established upto 6 months of age:
(1) Breast milk only (2) Formula milk only (3) Mixed

(4)Cow's milk (5) Goat's milk

28. Stopped breast feeding/non-breast feeding at neonatal period? 1=yes, 2=no

29. Why breast feeding was stopped at neonatal period?
1= Mother died during/after delivery or within neonatal period
2= Fostered from neonatal period

3= For medical contraindication from mothers side
4= Ignorance of care givers

30. Any illness of the patient since birth? 1=yes,2=no,9=NA

31. If yes, detail diagnosis (es) and treatment(s) with dates:
1=AWD, 2=ID, 3=PD, 4=Pneumonia, 5=PTB, 6=Sepsis, 7=Convulsion/Seizure,
8= childhood asthma, 9= chronic lung disease, 10= Others

32. Treatment given for that illness
1=Antibiotics, 2=Analgesics, 3=Bronchodilator, 4=Anti convulsant, 5=others

---

33. **Immunizations to date:**

BCG	1= Yes,	2=No , 3=Don't know, 9=NA
PENTA 1/oral polio	1= Yes,	2=No, 3=Don't know, 9=NA
PENTA 2/ oral polio 2	1= Yes,	2=No, 3=Don't know ,9=NA
PENTA 3/ oral polio 3	1= Yes,	2=No, 3=Don't know, 9=NA
Oral polio 4	1=Yes,	2=No, 3=Don't know, 9=NA
Hib 1st dose given	1= Yes,	2=No, 3=Don't know, 9=NA
Hib 2nd dose given	1= Yes,	2=No, 3=Don't know, 9=NA
Hib 3rd dose given	1=Yes,	2=No, 3=Don't know, 9=NA
MR	1= Yes,	2=No, 3=Don't know, 9=NA

34. History of contact with patient suffering from TB? 1= Yes, 2=No, 3= Don't know

35. If yes, relationship to child:
1=Paternal relatives, 2=Maternal relatives,3=Both, 4=Neighbour,
5= any of the parents, 6= any of the siblings, 7= Others

36. Type of TB of the contact:
1=PTB,Closed type, 2=PTB,Open type, 3= Extra pulmonary, 8=Don't know

37. When treated?
1=within last one month,2=within two to six months, 3=more than six months

38. Indoor smoke exposure:

Charcoal 1= Yes, 2=No

Kerosine 1= Yes, 2=No

Cigarette (inside room) 1= Yes, 2=No

Cigarette(outside room) 1=Yes, 2=No

39. Do you have a separate living room?

1=yes, 2=no

40. Is there any kitchen in the house?
1=yes, 2=no

41. How far is the kitchen from the living/bed rooms?
1= inside the bedroom, 2= another room,
3= open place outside the living room

42. Is there any window / exhaust fan in the cooking room?
1= yes, 2= no,3= open place, 4= Not applicable

SECTION 2: CLINICAL INFORMATION

In days

43. Symptoms:

Fever 1= yes, 2= no for how long? ______

Cough 1= yes, 2= no for how long? ______

Nasal discharge 1= yes, 2= no for how long ? ______

Diarrhoea 1= yes, 2= no for how long? ______ If yes, Clinical types of diarrhea 1=AWD, 2=ID, 3=PD

Dehydration 1= yes, 2= no

Clinical types of dehydration on admission 1=no sign, 2= some, 3= severe

Vomiting 1= yes, 2= no for how long? ______

Poor feeding 1= yes, 2= no for how long? ______

Difficulty in breathing 1= yes, 2= no for how long? ______

Oedema 1= yes, 2= no for how long? ______

Irritable 1= yes, 2= no for how long? ______

Rash 1= yes, 2= no for how long? ______

Convulsions 1= yes, 2= no for how long? ______

Number of episodes of convulsion prior to admission

Number of episodes of convulsion after admission

<u>Past medical history</u>:

44. Was there any previous admission? 1= yes, 2= no, 3=don't know

45. What was the reason of the previous admission? 1=Pneumonia, 2=PTB, 3=Asthma, 4=Foreign body aspiration,5=Diarrhea,6=Sepsis,7=Others

46. Did patient receive any antibiotics for this illness prior to admission?
    1=yes, 2=no, 3=Don't Know

47. Did the patient receive ampicillin/amoxicillin within 24 hours? 1=Yes, 2=No

48. Did the patient receive chloramphenicol? 1=Yes, 2=No

49. Did the patient receive azithromycin? 1=Yes, 2=No

50. Did the patient received amikacin? 1=Yes, 2=No

51. Did the patient receive ceftazidime? 1=Yes, 2=No

52. Did the patient receive gentamycin? 1=Yes, 2=No

53. Did the patient receive ceftriaxone? 1=Yes, 2=No

<u>Physical examinations</u>:

54. Weight: kg

Height/length: . cm

55. Z- score against weight for length/height: ------------.-----------

Z- score against weight for Age: -----------.---------------

56. MUAC : -----------.--------------- cm

57. Temperature (axilla):

58. Heart rate: bpm

59. Respiratory rate: ☐☐ bpm

(at rest ,when not crying or coughing):

60. BP: ______/_____ mmHg

61. Capillary refill time : ____ seconds

62. Skin rash: 1=yes, 2=No ☐

63. Characteristics of the skin rash on admission ☐
1=macular,2= papular,3=maculo-papular,4=petichieal,
5=purpuric, 6= scaly, 7= urticarial, 8=others

64. Pedal oedema: 1=0,2=+, 3=++ , 4=+++ ☐

65. Pallor: 1=0,2=+, 3=++ ,4=+++ ☐

66. Eye discharge: 1=yes,2=No ☐

67. Nasal discharge: 1=yes,2=No ☐

68. Ear discharge: Right 1=yes,2=No ☐

Left 1=yes,2=No ☐

69.ASOM: Right 1=yes,2=No ☐

Left 1=yes,2=No ☐

70. Oral thrush: 1=yes,2=No ☐

71. Lymphadenopathy: 1=yes,2=No ☐

72. If Yes, generalized 1=yes,2=No ☐

73. If generalized lymhadenopathy: symmetrical node enlargement >2cm in at least two different anatomical sites 1=yes,2=No ☐

74. Digital clubbing: 1=yes,2=No ☐

75. Parotid swelling: 1=yes,2=No ☐

<u>Respiratory findings</u>:

76. Lower chest wall indrawing 1=yes,2=No

77. Nasal flaring 1=yes,2=No

78. Central cyanosis 1=yes ,2=No

79. Grunting 1=yes ,2=No

80. Head nodding 1=yes ,2=No

81. Clear chest 1=yes ,2=No

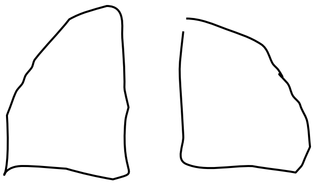

82. Crackles 1=yes,2=No

83. Wheeze/Ronchi 1=yes,2=No

84. Bronchial breath sounds 1=yes,2=No

Abdominal exam:

85. Distended abdomen 1=yes , 2=No

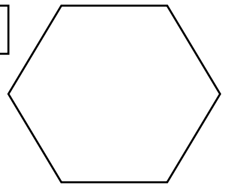

86. Liver edge below RCM __ cm

87. Spleen edge below CM __ cm

88. Mental state:

Is the child Alert 1=yes, 2=No

Is the child Irritable 1=yes, 2=No

Is the child Lethargic 1=yes, 2=No

Response to vocal command 1=yes, 2=No

Response to pain stimuli 1=yes, 2=No

Patient is unconscious 1=yes, 2=No

Level of consciousness: GCS __

89. Additional information on admission:

1=umbilical hernia , 2=Visual impairment or hearing loss, 3=others

90. Oxygen saturation on admission in air _____ %

91. PH on admission

92. PCO2 on admission

93. PO2 on admission

94. Base excess on admission

95. TCO2 on admission

96. HCO3 on admission

97. Random blood sugar : _____._____ mmol/L

98**.** Main clinical diagnosis:
1. Very severe pneumonia
2. Very severe pneumonia with sepsis
3. very sever pneumonia withsevere sepsis
4. very sever pneumonia with septic shock
5. Other

Physician's initials: ____________ Date: ___/___/____

Principal investigator will complete final diagnosis according to study codes:

Final clinical diagnosis :

SECTION 3: INVESTIGATIONS AND MANAGEMENT

99. Hypoglycaemia on or after admission (<3 mmol/L) 1=Yes, [illegible]o

100. Total WBC/cu.mm (Not done= 99)

101. Poly(%) (Not done= 99)

102. Lymphocytes (%) (Not done= 99)

103. Immature poly (Band) % (Not done= 99)

104. Hct% (Not done= 99)

105. Sodium, mol/L (Not done= 99)

106. Potassium; mmol/L (Not done= 99)

107. Chloride; mmol/L (Not done= 99)

108. TCO_2; mmol/L (Not done= 99)

109. Creatinine; micromol/L (Not done= 99)

110. Calcium; micromol/L (Not done= 99)

111 .Magnesium; micromol/L (Not done= 99)

Examination after initiation of treatment on D1

112. Severe hypoxaemia (SpO_2<85%) 1=yes, 2=No

113.Exhaustion 1=yes, 2=No

114.Active contraction of respiratory muscles 1=yes,2=No

115.Paradoxical abdominal and thoracic motion 1=yes,2=No

116.Gasping 1=yes,2=No

117. Severe lower chest wall indrawing 1=yes,2=No

118.Grunting respiration 1=yes,2=No

119.Patient is on ventilator 1=yes,2=No

Examination after initiation of treatment on D2

120.Severe hypoxaemia (SpO_2<85%) 1=yes, 2=No

121.Exhaustion 1=yes, 2=No

122.Active contraction of respiratory muscles 1=yes, 2=No

123.Paradoxical abdominal and thoracic motion 1=yes, 2=No

124.Gasping 1=yes, 2=No

125. Severe lower chest wall indrawing 1=yes, 2=No

126.Grunting respiration 1=yes, 2=No

127.Patient is on ventilator 1=yes, 2=No

Examination after initiation of treatment on D3

128.Severe hypoxaemia (SpO_2<85%) 1=yes, 2=No

129.Exhaustion 1=yes,2=No

130.Active contraction of respiratory muscles 1=yes,2=No

131.Paradoxical abdominal and thoracic motion 1=yes,2=No

132.Gasping 1=yes,2=No

133. Severe lower chest wall indrawing 1=yes,2=No

134.Grunting respiration 1=yes,2=No

135.Patient is on ventilator 1=yes, 2=No

Microbiology

136. Blood culture: 1=Positive, 2= Negative, 3=Unknown, 4=Not done

137. **Blood culture isolate** :
1=Streptococcus pneumoniae, 2=staphylococcus aureus,
3=Haemophylus influenzae, 4=Salmonalla typhi, 5=Salmonalla para-typhi,
6=Non-typhoidal Salmonella, 7=E. Coli, 8=Klebseilla, 9= Pseudomonas spp/auregenosa
10= Acenetobacter, 11=CNS

138. **Gastric lavage for AFB :**
1=+ve for AFB, 2=-ve for AFB, 3=not done,
4=Pt's Died before procedure, 5=Patient absconded

139. **Gastric lavage for CS** :
1= +ve for MTB, 2= +ve for non-tubrercular mycobacteria,
3= no growth of mycobacteria, 4= could not be sent

140. **Chest x-ray** : 1. lobar consolidation, 2. patchy consolidation, 3=discrete opacities
4. Normal x-Ray

141. Antibiotics given:

Antibiotic and dose	1=yes, 2=No	Day started (dd/mm/yy)	Day stopped (dd/mm/yy)	comment
Ampicilin 100mg/kg.qid				

Gentamicin 5mg/kg od				
Ceftriaxone 100mg/kg/od				
Levofloxacin 10mg/kg/dose				
Ceftazidime 75-100mg/kg tds				
Amikacin 15mg/kg/dose bid/ tds				
Flucloxacillin 50mg/kg/day qid				
Others(1=meropenum/2=imipenum/ 3=vancomycin/4=anti-PCJP)				

142. Did patient receive any Inotropes ? 1=yes,2=No

1. Did the patient receive Dopamin ? 1=yes, 2=No, 9= Not applicable
2. Did the patient receive Adrenalin ? 1=yes, 2=No, 9= Not applicable
3. Did the patient receive Noradrenalin ? 1=yes, 2=No, 9= Not applicable
4. Did the patient receive Atropin? 1=yes, 2=No, 9= Not applicable

143. Did patient develop Heart failure ? 1=yes,2=No

1. Did the patient receive Frusimide? 1=yes, 2=No, 9= Not applicable
2. Did the patient receive Digoxin? 1=yes, 2=No, 9= Not applicable

144. Did patient receive any Blood transfusion ? 1=yes,2=No

145. **Oxygen**

Day started (Day/Month/Year) ______/_______/________ at: ______: ______ AM/PM

Day stopped (Day/Month/Year) ______/_____/______ ____ at: ______: ______ AM/PM

146.Total Duration of oxygen supplementation in hours [(1) bubble CPAP, (2) the standard O_2 supplementation by nasal prongs , (3) humidified high flow nasal cannula]: Hours

Other Information:

147.Duration of normalization of respiratory rate. Hrs

148.Duration of disappearance of chest wall indrawing Hrs

149.Duration of disappearance of dangers sign (s) (if any). Hrs

150. Nosocomial infections 1=yes,2=No

SECTION 4: OUTCOME AND FOLLOW-UP

151. Final Diagnosis:

1. Bacterial pneumonia 1=yes,2=No
2. PCJP 1=yes,2=No
3. PTB on the basis of MKJ scoring / WHO scoring 1=yes,2=No
4. PTB on the basis of AFB in gastric lavage 1=yes,2=No
5. PTB on the basis of positive gastric lavage culture 1=yes,2=No
6. Aetiology unknown 1=yes,2=No

152. Final Outcome**:**

1. Well & discharged
2. Improved but still hypoxaemic on discharge
3. Did not improve but discharged on request
4. Died in hospital
5. Died at home
6. DORB
7. Absconded

153. Did the patient develop "Treatment failure" from any arm?

154. If death, cause of death:
1=Severe sepsis, 2= Septic shock, 3= Meningitis/encephalitis, 4=MODS, 5=Neonatal Sepsis, 6= severe diselectrolytaimia, 7= SAM, 9= not applicable

155. Treatment on discharge:

1. Antibiotic course (e.g. amoxycillin, flucloxacillin, c'col etc)
2. TB treatment
3. Micronutrients

Physician's initials: ________________________ Date: ____/____/______

Annex 5 Gender Analysis Framework:

Efficacy of two doses of parenteral Amoxicillin plus single dose Gentamicin compared to four doses of parenteral Ampicillin plus single dose Gentamicin in managing children under five with severe pneumonia: an open labelled randomized controlled clinical trial

**In Relation to Severe pneumonia**	**Are there sex differences in**	**How do biological differences between women and men influence their :**	**How do the different roles and activities of men and women affect their:**	**How do gender norms / values affect men and women's**	**How do access to, and control over resources affect men and women's**
**Vulnerability:**<br><br>**Incidence ****<br>**Prevalence ****<br>**(male/female)**	*No sex difference found in any published literature. However after completion of this study we will have a overview of the prevalence of severe pneumonia*	*Not applicable because biological differences do not make someone more hypernatremic.*	*No role yet found*	*Not applicable*	*As it is a hospital based study access and control over resources will be according to the hospital existing Policy*
**Health seeking behaviour**	*No studies yet found any difference in this context*	*Not applicable*	*Although there is common practise in society to give less attention to female children, but in this hospital based controlled setting this issue will be averted*	*No gender norms in Same as above Severe pneumonia*	
**Ability to access health services**	Access to health services is among different sex group has been minimized				
**Experience with health services and health providers**	*No published literature has shown any difference i this issue however according to current hospital practise in icddr,b all patient will receive equal health service and there will be no gender norms*				
**Preventive and Treatment options, responses to treatment or rehabilitation**	*Not related to this study*				

In Relation to Severe pneumonia	Are there sex differences in	How do biological differences between women and men influence their :	How do the different roles and activities of men and women affect their:	How do gender norms / values affect men and women's	How do access to, and control over resources affect men and women's
**Outcome of health problem**	There is evident in different sex in outcome	*Not applicable*	*No published literature has shown any difference in outcome , gender norms will not be affected in this hospital setting of severe pneumonia management*		
**Consequences (economic & social, including attitudinal)**	*Not related*	*Not known*	*Not yet been addressed*		

Protocol Title: Efficacy of two doses of parenteral Amoxicillin plus single dose Gentamicin compared to four doses of parenteral Ampicillin plus single dose Gentamicin in managing children hospitalized with WHO classified severe pneumonia: an open labelled randomized controlled clinical trial

Investigator's name: Dr Lubaba Shahrin

Organization: International Centre for Diarrhoeal Disease and Research, Bangladesh

Purpose of the research

Severe pneumonia is a serious infection in children which requires timely intervention. Management of severe pneumonia is done by appropriate antibiotics and supportive care. WHO recommends using injection Ampicillin four times a day for 3-5 days and injection Gentamicin once a day for 3-5 days in children with severe pneumonia. In our study we want to compare the response of injection Ampicillin with another similar and equally effective antibiotic (amoxicillin). The reason of choosing amoxicillin is that the dose is two times a day and it is cost effective and reduces chance of nosocomial infection. The response of the drugs will be compared by time difference of clinical improvement of the patients or time difference of disappearance of the sign and symptoms on admission. We are interested to compare the recovery time from the illness as well as treatment failure in both group. In that way we will be able to develop a better treatment for pneumonia in children.

Why invited to participate in the study?

As we are comparing the efficacy of two treatment regimen in the children with severe pneumonia, we have to enroll hospitalized patient. Your child has been identified because the child is admitted to hospital with severe pneumonia. We are doing this research to help to identify better ways to care for children in the future.

Please feel free to ask any questions you may have about this study and the information given below. You will be given opportunity to ask questions will be answered. If you agree to our proposal of inclusion of your child in our study, we would provide you with a copy of this consent form.

Methods and procedures

If you agree to our proposal of including you in the study then:

- Your child will continue to receive usual care and treatment for the condition with which he/she is admitted
- We will review the medical records from your child's hospitalization, previous hospitalizations and hospitalizations that occur during study follow-up.
- We will perform detailed measurements of your child's nutritional status and food intake.
- If required, we may perform a chest x ray to your child, which is non invasive and with minimum hazard.
- If your child is sick we may require taking 5 mL of blood sample (approximately 1 tea spoon full) according to usual hospital management protocol.
- After the study has finished we may wish to contact you again, to ask if you are free of illness after discharge or not. We will gather some additional information about your household and socio-economic status.
- For the purpose of the study our research staff may take your photograph.

Risk and benefits

Some pain and discomfort will be experienced from the needle prick when blood samples are obtained. Very occasionally, taking blood samples might cause infection. Careful attention will however be paid to ensure that the sample is taken in a professional manner to avoid these problems. The benefits for your child taking part in this study will be extensive monitoring during whole hospitalization period, free of cost treatment, other logistic support and free medical consultation even after discharge from the study. The results of this study will be beneficial to society in the future as they may help improve care for children with malnutrition.

Privacy, anonymity and confidentiality

We shall assure that the privacy, anonymity and confidentiality of data/information identifying your patient will be strictly maintained. We would keep all the medical information, description of treatment, and results of laboratory tests performed on your patient confidential, and stored in a safe place, under lock and key under the responsibility of the principal investigator. None other than the investigators of this research study; possible study monitor; regulatory authorities, such as the Ethical Review Committee (ERC) of icddr,b; and any law-enforcing agency in the event of necessity would have an access to the information. We want to inform you that data related

to the study will not be sent outside the country for analysis. The name or identity of your child would not be disclosed while publishing the results of this study.

Future use of information

In case of future use of the information collected from the study, the investigators will arrange to provide that in such cases anonymous or abstracted information and data may be supplied to other researchers, which should not conflict with or violate the maintenance of privacy, anonymity and confidentiality of information identifying participants in any way.

The chest x ray will be preserved for study purpose up to 5 years after completion of the study. Blood will be stored for any future molecular analysis to identify the causative organism.

Right not to participate and withdraw

Participation in research is voluntary. You are free to decide if you want your child to take part in research or not. Your child will still receive the recommended standard care whether or not you agree to take part. If you have decided to participate, you can change your mind any time and withdraw your child from the study. This will not affect your child's care now or in the future.

Principle of compensation

For any study related injuries, we will provide treatment in the hospital at free of cost.

Answering your questions/ Contact persons

You can ask any of our workers anytime. You can also communicate with those involved in taking care of your child and this study. At any point of time during hospitalization and even after discharge you can contact with the PI (Dr Lubaba Shahrin) for any health related query of your child.

If you agree to our proposal of enrolling you/your patient in our study, please indicate that by putting your signature or your left thumb impression at the specified space below

Thank you for your cooperation.

______________________________ ______________

Signature or left thumb impression of participant's legal guardian Date

______________________________ ______________

Signature or left thumb impression of the witness Date

______________________________ ______________

Signature of the PI or his/her representative Date

(NOTE: In case of representative of the PI, she/he shall put her/his full name and designation and then sign)

(Name and contact phone of IRB Secretariat, RA, M. A. Salam Khan, Phone No: 9886498 or PABX 8860523-32 Extension. 3206).

গবেষণার নামঃ **<u>Efficacy of two doses of parenteral Amoxicillin plus single dose Gentamicin compared to four doses of parenteral Ampicillin plus single dose Gentamicin in managing children hospitalized with WHO classified severe pneumonia: an open labeled randomized controlled clinical trial</u>**

গবেষকের নামঃ ডাঃ লুবাবা শাহরিন

প্রতিষ্ঠানের নামঃ আন্তর্জাতিক উদরাময় গবেষণা কেন্দ্র, বাংলাদেশ

গবেষণার উদ্দেশ্যঃ

মারাত্মক নিউমোনিয়া শিশুদের জন্য একটি জীবনঘাতী সংক্রমন এবং এর জন্য সময়োপযোগী চিকিৎসা দেয়া খুবই জরুরী। এর চিকিৎসা উপযুক্ত এন্টিবায়োটিক ও সহায়ক তত্ত্বাবধানের মাধ্যমে করা হয়। বিশ্ব স্বাস্থ্য সংস্থার নির্দেশ হচ্ছে দিনে ৪ বার এম্পিসিলিন ইঞ্জেকশন ও ১ বার জেন্টামাইসিন ইঞ্জেকশন এই দুইটি ওষুধ ৩-৫ দিন ব্যবহার করা। আমাদের গবেষণায় এই এম্পিসিলিনের চিকিৎসা পদ্ধতির সাথে আরেকটি অনুরূপভাবে কার্যকর এন্টিবায়োটিক (এমোক্সিসিলিন) এর ব্যবহারে চিকিৎসা পদ্ধতির তুলনা করে দেখতে চাই। এমোক্সিসিলিন নির্বাচনের কারণ হচ্ছে এটা দিনে দুইবার ব্যবহার করতে হয়, এটি সাশ্রয়ী এবং এটি হাসপাতাল সংক্রমনের ঝুঁকি কমায়। রোগীর উন্নতি হতে, বা রোগের লক্ষণ দূর হতে প্রয়োজনীয় সময় এর তুলনা করে আমরা এই দুইটি চিকিৎসা পদ্ধতির তুলনা করবো। পাশাপাশি আরোগ্য লাভের সময় এবং চিকিৎসায় ব্যর্থতার হার এই দুইটি বিষয়ও আমরা তুলনা করে দেখতে চাই। এর মাধ্যমে আমরা শিশুদের জন্য একটি শ্রেয়তর চিকিৎসা পদ্ধতি নির্ণয় করতে পারবো।

কেন এই গবেষণায় অংশগ্রহনের আমন্ত্রণ জানানো হয়েছে?

যেহেতু আমরা মারাত্মক নিউমোনিয়ার চিকিৎসায় দুটি চিকিৎসা পদ্ধতির তুলনামূলক বিচার করছি, তাই এই গবেষণায় হাসপাতালে ভর্তি রোগীদের অন্তর্ভূক্ত করতে হবে। আপনার শিশুকে নির্বাচন করা হয়েছে কারন সে মারাত্মক নিউমোনিয়া নিয়ে এই হাসপাতালে ভর্তি হয়েছে। এই গবেষণা ভবিষ্যতে শিশুদের জন্য উন্নত চিকিৎসা নির্ধারনে ভূমিকা রাখবে।

আপনাকে এই সম্মতিপত্রে যে তথ্য দেয়া হবে, তার সম্পর্কে বা এই গবেষণার সম্পর্কে আপনার কোন প্রশ্ন থাকলে আমাদের নির্দ্বির্ধায় জিজ্ঞেস করতে পারেন। আপনাকে প্রশ্ন করার সুযোগ দেয়া হবে এবং আপনার প্রশ্নের উত্তর দেয়া হবে। আপনি যদি আমাদের প্রস্তাবে রাজি হয়ে এই গবেষণায় আপনার শিশুকে অংশগ্রহন করতে দিতে সম্মত হন, তাহলে আপনাকে এই সম্মতি পত্রের একটি অনুলিপি দেয়া হবে।

গবেষণার পদ্ধতি ও প্রক্রিয়াঃ

যদি আপনি আমাদের প্রস্তাবে সম্মত হয়ে এই গবেষণায় আপনার সন্তানকে অন্তর্ভূক্ত করেন তাহলে আপনি নিম্নলিখিত কর্মকান্ডগুলি আশা করতে পারেনঃ

- আপনার শিশু তার অসুস্থতার জন্য সাধারন চিকিৎসা সেবা পাবে।
- আমরা তার চিকিৎসার বিবরন পর্যবেক্ষন করবো।
- আমরা তার পুষ্টির অবস্থা ও খাদ্য গ্রহনের পরিমাপ করবো
- প্রয়োজনে আমরা তার বক্ষদেশের একটি এক্স রে করাতে পারি, যেটি শিশুর কোন ক্ষতি করবে না
- যদি আপনার শিশু বেশী অসুস্থ হয়, সেক্ষেত্রে আমরা তার কাছ থেকে <u>৫ মিঃলিঃ (১ চা চামচের সমান)</u> রক্ত নমুনা সংগ্রহ করতে পারি। হাসপাতালের স্বাভাবিক নিয়ম মেনেই এই রক্ত সংগ্রহ করা হবে।
- গবেষনার শেষে আমরা পুনরায় আপনার সাথে যোগাযোগ করে জিজ্ঞেস করতে পারি আপনার শিশু অসুস্থ না সুস্থ অবস্থায় আছে। একই সাথে এই সময় আপনার বাসা ও আর্থসামাজিক অবস্থা সম্পর্কে কিছু প্রশ্ন করা হবে।
- গবেষণার প্রয়োজনে আমাদের গবেষণা কর্মী আপনাদের ছবি তুলতে পারে।

গবেষণার ঝুঁকি ও এর সাথে জড়িত সুবিধাঃ

সুই প্রবেশ করিয়ে রক্ত সংগ্রহ করার সময় সামান্য ব্যাথা বা অস্বস্তি অনুভূত হতে পারে। কদাচিৎ রক্ত সংগ্রহের কারনে সংক্রমনের সম্ভাবনা থাকে। এর জন্য যথাসম্ভব ব্যবস্থা নেয়া হবে। এই গবেষণায় অংশগ্রহনের ফলে আপনার শিশু আমাদের হাসপাতালের নিবিড় তত্ত্বাবধানে থাকবে। এছাড়া এর ফলে, গবেষণা শেষ হয়ে যাওয়ার পরেও সে বিনামূল্যে চিকিৎসা সেবা ও পরামর্শ পাবে। এই গবেষণায় প্রাপ্ত তথ্য সমাজে শিশুদের কার্যকর চিকিৎসার উন্নতি সাধনে ভূমিকা রাখবে।

গোপনীয়তা, নামহীনতা ও বিশ্বস্ততাঃ

আমরা আপনাকে এই মর্মে আশ্বস্ত করতে চাই যে, যে সকল তথ্য দিয়ে আপনাকে বা আপনার শিশুকে সনাক্ত করা যায় তা অত্যন্ত গোপনীয়তার সাথে রক্ষা করা হবে। আপনার শিশুর স্বাস্থ্য ও চিকিৎসা বিষয়ক সকল তথ্য ও সকল পরীক্ষা নিরীক্ষার ফলাফল গোপনীয় রাখা হবে। গবেষনার গবেষকবৃন্দ ছাড়া আর কারো এই তথ্য দেখা অধিকার থাকবে না। গবেষনার তথ্য বিশ্লেষনের প্রয়োজনে বিদেশে পাঠানো যেতে পারে। কিন্তু আপনাকে ব্যক্তিগতভাবে সনাক্ত করা যায় এমন সকল তথ্য সুসংরক্ষিত থাকবে এবং নিরাপত্তার সাথে প্রক্রিয়াভুক্ত করা হবে। আমাদের প্রতিষ্ঠানের সীমিত সংখ্যক কর্মীরই এই তথ্য দেখার অধিকার থাকবে। গবেষণার ফলাফল প্রকাশের সময় আপনার শিশুর নাম বা পরিচয় প্রকাশ করা হবে এমন সম্ভাবনা নেই।

ভবিষ্যতে তথ্যের ব্যবহারঃ
যদি এই গবেষনার কোন তথ্য বা জৈবিক নমুনা পরবর্তীতে কোন কাজে ব্যবহার করা হয়, তাহলে আমরা আপনাকে আশ্বস্ত করতে চাই যে, সে সময়ও বেনামী বা সংক্ষিপ্তকৃত তথ্য এবং অন্যান্য গবেষকদের সরবরাহ করা হতে পারে, যা কোনভাবেই অংশগ্রহনকারীদের পরিচিতি গোপন রাখার জন্য যে গোপনীয়তা ও বিশ্বস্ততার প্রতিশ্রুতি দেয়া হয়েছে তার বিরোধিতা করবে না। গবেষনায় যে এক্স রে গুলো করা হবে, তার গবেষনার পরবর্তী ৫ বছর পর্যন্ত সংরক্ষণ করা হবে। রক্তের নমুনা সংরক্ষন করা হবে, যা ভবিষ্যতে রোগ সৃষ্টিকারী অণুজীব সনাক্ত করার জন্য আণবিক বিশ্লেষণ এর জন্য ব্যবহৃত হতে পারে।
গবেষণায় অংশগ্রহন না করা ও নাম প্রত্যাহারের অধিকারঃ
এই গবেষনায় আপনার অংশগ্রহন সম্পূর্ণ ঐচ্ছিক, এবং আপনার সন্তানকে গবেষনায় অংশগ্রহন করতে দেয়ার চূড়ান্ত সিদ্ধান্ত শুধুমাত্র আপনিই সংরক্ষন করেন। আপনি গবেষণায় আপনার শিশুকে অন্তর্ভূক্ত না করলেও হাসপাতাল থেকে আপনার শিশু তার প্রাপ্য মানসম্মত চিকিৎসা সেবা পাবে। এছাড়াও, গবেষনার যে কোন পর্যায়ে আপনি এ থেকে নাম প্রত্যাহার করে নিতে পারেন। গবেষনায় অংশগ্রহন না করলে/নাম প্রত্যাহার করে নিলে আপনি কোন সুবিধা থেকে বঞ্চিত হবেন না।
ক্ষতিপূরণের মূলনীতিঃ
এই গবেষনায় অংশগ্রহনকালে কোন অসুখ এর জন্য আপনার শিশুকে সর্বোত্তম চিকিৎসা সেবা প্রদান করা হবে।

প্রশ্নের উত্তরের জন্য যোগাযোগের ব্যক্তিঃ
আমাদের কর্মীরা যে কোন সময় আপনার প্রশ্নের উত্তর দিতে প্রস্তুত থাকবে। আপনি আপনার শিশুকে সেবাদানকারীদেরও জিজ্ঞেস করতে পারেন। এছাড়া হাসপাতালে থাকাকালীন সময়ে বা হাসপাতাল থেকে চলে যাওয়ার পরেও আপনার শিশুর স্বাস্থ্য বিষয়ক কোন জিজ্ঞাস্য থাকলে আপনি এই গবেষণার প্রধান গবেষক ডাঃ লুবাবা শাহরিন এর সাথে যোগাযোগ করতে পারেন।

যদি আপনি আমাদের প্রস্তাবে রাজি হয়ে এই গবেষণায় অংশগ্রহনের সম্মতি প্রদান করেন, তাহলে নিচে আপনার স্বাক্ষর বা টিপসই দিয়ে তা প্রকাশ করুন।
আপনার সহযোগিতার জন্য ধন্যবাদ।

অংশগ্রহনকারীর অভিভাবক এর স্বাক্ষর বা টিপসই   তারিখ

সাক্ষীর স্বাক্ষর বা টিপসই   তারিখ

প্রধান গবেষক বা তার প্রতিনিধির স্বাক্ষর   তারিখ

বিঃদ্রঃ প্রধান গবেষকের প্রতিনিধি তার পূর্ণ নাম ও পদবী লিখে তারপর স্বাক্ষর করবেন।

(আই, আর, বি দপ্তরের যোগাযোগের নাম ও ঠিকানাঃ জনাব এম, এ, সালাম খান, ফোনঃ ৯৮৮৬৪৯৮ বা ৯৮২৭০০১-১০, এক্সটঃ ৩২০৬)
