# Supplementary material for: Injectable Amoxicillin Versus Injectable Ampicillin Plus Gentamicin in the Treatment of Severe Pneumonia in Children Aged 2 to 59 Months: Protocol for an Open-Label Randomized Controlled Trial
Source: JMIR Res Protoc. 2020 Nov 2;9(11):e17735. doi: 10.2196/17735 (PMC7669443; doi:10.2196/17735)
Supplement: Multimedia Appendix 3 [file resprot_v9i11e17735_app3.pdf]

## ঐচ্ছিক সম্মতি পত্র

| গবেষণা নং: চজ-১৭০৬১ | ভাষান্তর নং: ১.৩ | তারিখঃ ১২-০৮-২০১৭ |
|---|---|---|

**M‡elYvi bvgt Efficacy of two doses of parenteral Amoxicillin plus single dose Gentamicin compared to four doses of parenteral Ampicillin plus single dose Gentamicin in managing children hospitalized with WHO classified severe pneumonia: an open labeled randomized controlled clinical trial**

**গবেষকের নামঃ** ডাঃ লুবাবা শাহরিন

**প্রতিষ্ঠানের নামঃ** আন্তর্জাতিক উদরাময় গবেষণা কেন্দ্র, বাংলাদেশ

**গবেষণার উদ্দেশ্যঃ**

মারাত্মক নিউমোনিয়া শিশুদের জন্য একটি জীবনঘাতী সংক্রমন এবং এর জন্য সময়োপযোগী চিকিৎসা দেয়া খুবই জরুরী। এর চিকিৎসা উপযুক্ত এন্টিবায়োটিক ও সহায়ক তত্ত্বাবধানের মাধ্যমে করা হয়। বিশ্ব স্বাস্থ্য সংস্থার নির্দেশ হচ্ছে দিনে ৪ বার এম্পিসিলিন ইঞ্জেকশন ও ১ বার জেন্টামাইসিন ইঞ্জেকশন এই দুইটি ওষুধ ৩-৫ দিন ব্যবহার করা। আমাদের গবেষণায় এই এম্পিসিলিনের চিকিৎসা পদ্ধতির সাথে আরেকটি অনুরূপভাবে কার্যকর এন্টিবায়োটিক (এমোক্সিসিলিন) এর ব্যবহারে চিকিৎসা পদ্ধতির তুলনা করে দেখতে চাই। এমোক্সিসিলিন নির্বাচনের কারণ হচ্ছে এটা দিনে দুইবার ব্যবহার করতে হয়, এটি সাশ্রয়ী এবং এটি হাসপাতাল সংক্রমনের ঝুঁকি কমায়। রোগীর উন্নতি হতে, বা রোগের লক্ষণ দূর হতে প্রয়োজনীয় সময় এর তুলনা করে আমরা এই দুইটি চিকিৎসা পদ্ধতির তুলনা করবো। পাশাপাশি আরোগ্য লাভের সময় এবং চিকিৎসায় ব্যথতার হার এই দুইটি বিষয়ও আমরা তুলনা করে দেখতে চাই। এর মাধ্যমে আমরা শিশুদের জন্য একটি শ্রেয়তর চিকিৎসা পদ্ধতি নির্ণয় করতে পারবো।

**কেন এই গবেষণায় অংশগ্রহনের আমন্ত্রণ জানানো হয়েছে?**

যেহেতু আমরা মারাত্মক নিউমোনিয়ার চিকিৎসায় দুটি চিকিৎসা পদ্ধতির তুলনামূলক বিচার করছি, তাই এই গবেষণায় হাসপাতালে ভর্তি রোগীদের অন্তর্ভূক্ত করতে হবে। আপনার শিশুকে নির্বাচন করা হয়েছে কারন সে মারাত্মক নিউমোনিয়া নিয়ে এই হাসপাতালে ভর্তি হয়েছে। এই গবেষণা ভবিষ্যতে শিশুদের জন্য উন্নত চিকিৎসা নির্ধারনে ভূমিকা রাখবে।

আপনাকে এই সম্মতিপত্রে যে তথ্য দেয়া হবে, তার সম্পর্কে বা এই গবেষণার সম্পর্কে আপনার কোন প্রশ্ন থাকলে আমাদের নিদ্বির্ধায় জিজ্ঞেস করতে পারেন। আপনাকে প্রশ্ন করার সুযোগ দেয়া হবে এবং আপনার প্রশ্নের উত্তর দেয়া হবে। আপনি যদি আমাদের প্রস্তাবে রাজি হয়ে এই গবেষণায় আপনার শিশুকে অংশগ্রহন করতে দিতে সম্মত হন, তাহলে আপনাকে এই সম্মতি পত্রের একটি অনুলিপি দেয়া হবে।

**গবেষণার পদ্ধতি ও প্রক্রিয়াঃ**

যদি আপনি আমাদের প্রস্তাবে সম্মত হয়ে এই গবেষণায় আপনার সন্তানকে অন্তর্ভূক্ত করেন তাহলে আপনি নিম্নলিখিত কর্মকান্ডগুলি আশা করতে পারেনঃ

- আপনার শিশু তার অসুস্থতার জন্য সাধারন চিকিৎসা সেবা পাবে।
- আমরা তার চিকিৎসার বিবরন পর্যবেক্ষন করবো।
- আমরা তার পুষ্টির অবস্থা ও খাদ্য গ্রহনের পরিমাপ করবো
- প্রয়োজনে আমরা তার বক্ষদেশের একটি এক্স রে করাতে পারি, যেটি শিশুর কোন ক্ষতি করবে না

- যদি আপনার শিশু বেশী অসুস্থ হয়, সেক্ষেত্রে আমরা তার কাছ থেকে ৫ মিঃলিঃ (১ চা চামচের সমান) রক্ত নমুনা সংগ্রহ করতে পারি। হাসপাতালের স্বাভাবিক নিয়ম মেনেই এই রক্ত সংগ্রহ করা হবে।
- গবেষনার শেষে আমরা পুনরায় আপনার সাথে যোগাযোগ করে জিজ্ঞেস করতে পারি আপনার শিশু অসুস্থ না সুস্থ অবস্থায় আছে। একই সাথে এই সময় আপনার বাসা ও আর্থসামাজিক অবস্থা সম্পর্কে কিছু প্রশ্ন করা হবে।

**গবেষণার ঝুঁকি ও এর সাথে জড়িত সুবিধাঃ**

সুই প্রবেশ করিয়ে রক্ত সংগ্রহ করার সময় সামান্য ব্যাথা বা অস্বস্তি অনুভূত হতে পারে। কদাচিৎ রক্ত সংগ্রহের কারনে সংক্রমনের সম্ভাবনা থাকে। এর জন্য যথাসম্ভব ব্যবস্থা নেয়া হবে। এই গবেষণায় অংশগ্রহনের ফলে আপনার শিশু আমাদের হাসপাতালের নিবিড় তত্ত্বাবধানে থাকবে। এছাড়া এর ফলে, গবেষণা শেষ হয়ে যাওয়ার পরেও সে বিনামূল্যে চিকিৎসা সেবা ও পরামর্শ পাবে। এই গবেষণায় প্রাপ্ত তথ্য সমাজে শিশুদের কার্যকর চিকিৎসার উন্নতি সাধনে ভূমিকা রাখবে।

**গোপনীয়তা, নামহীনতা ও বিশ্বস্ততাঃ**

আমরা আপনাকে এই মর্মে আশ্বস্ত করতে চাই যে, যে সকল তথ্য দিয়ে আপনাকে বা আপনার শিশুকে সনাক্ত করা যায় তা অত্যন্ত গোপনীয়তার সাথে রক্ষা করা হবে। আপনার শিশুর স্বাস্থ্য ও চিকিৎসা বিষয়ক সকল  তথ্য ও সকল পরীক্ষা নিরীক্ষার ফলাফল গোপনীয় রাখা হবে। গবেষনার গবেষকবৃন্দ ছাড়া আর কারো এই তথ্য দেখা অধিকার থাকবে না। গবেষনার তথ্য বিশেস্নষনের প্রয়োজনে বিদেশে পাঠানো যেতে পারে। কিন্তু আপনাকে ব্যক্তিগতভাবে সনাক্ত করা যায় এমন সকল তথ্য সুসংরক্ষিত থাকবে এবং নিরাপত্তার সাথে প্রক্রিয়াভুক্ত করা হবে। আমাদের প্রতিষ্ঠানের সীমিত সংখ্যক কর্মীরই এই তথ্য দেখার অধিকার থাকবে। গবেষণার ফলাফল প্রকাশের সময় আপনার শিশুর নাম বা পরিচয় প্রকাশ করা হবে এমন সম্ভাবনা নেই।

**ভবিষ্যতে তথ্যের ব্যবহারঃ**

যদি এই গবেষনার কোন তথ্য বা জৈবিক নমুনা পরবর্তীতে কোন কাজে ব্যবহার করা হয়, তাহলে আমরা আপনাকে আশ্বস্ত করতে চাই যে, সে সময়ও বেনামী বা সংক্ষিপ্তকৃত তথ্য এবং অন্যান্য গবেষকদের সরবরাহ করা হতে পারে, যা কোনভাবেই অংশগ্রহনকারীদের পরিচিতি গোপন রাখার জন্য যে গোপনীয়তা ও বিশ্বস্ততার প্রতিশ্রম্নতি দেয়া হয়েছে তার বিরোধিতা করবে না। গবেষনায় যে এক্স রে গুলো করা হবে, তার গবেষনার পরবর্তী ৫ বছর পর্যন্ত সংরক্ষণ করা হবে। রক্তের নমুনা সংরক্ষন করা হবে, যা ভবিষ্যতে রোগ সৃষ্টিকারী অণুজীব সনাক্ত করার জন্য আণবিক বিশেস্নষণ এর জন্য ব্যবহৃত হতে পারে।

**গবেষণায় অংশগ্রহন না করা ও নাম প্রত্যাহারের অধিকারঃ**

এই গবেষনায় আপনার অংশগ্রহন সম্পূর্ণ ঐচ্ছিক, এবং আপনার সন্তানকে গবেষনায় অংশগ্রহন করতে দেয়ার চূড়ান্ত সিদ্ধান্ত শুধুমাত্র আপনিই সংরক্ষন করেন। আপনি গবেষণায় আপনার শিশুকে অর্ন্তভূক্ত না করলেও হাসপাতাল থেকে আপনার শিশু তার প্রাপ্য মানসম্মত চিকিৎসা সেবা পাবে। এছাড়াও, গবেষনার যে কোন পর্যায়ে আপনি এ থেকে নাম প্রত্যাহার করে নিতে পারেন। গবেষনায় অংশগ্রহন না করলে/নাম প্রত্যাহার করে নিলে আপনি কোন সুবিধা থেকে বঞ্চিত হবেন না।

**ক্ষতিপূরণের মূলনীতিঃ**

এই গবেষনায় অংশগ্রহনকালে কোন অসুখ এর জন্য আপনার শিশুকে সর্বোত্তম চিকিৎসা সেবা প্রদান করা হবে।

**প্রশ্নের উত্তরের জন্য যোগাযোগের ব্যক্তিঃ**

আমাদের কর্মীরা যে কোন সময় আপনার প্রশ্নের উত্তর দিতে প্রস্তুত থাকবে। আপনি আপনার শিশুকে সেবাদানকারীদেরও জিজ্ঞেস করতে পারেন। এছাড়া হাসপাতালে থাকাকালীন সময়ে বা হাসপাতাল থেকে চলে যাওয়ার পরেও আপনার শিশুর স্বাস্থ্য বিষয়ক কোন জিজ্ঞাস্য থাকলে আপনি এই গবেষণার প্রধান গবেষক ডাঃ লুবাবা শাহরিন এর সাথে যোগাযোগ করতে পারেন।

যদি আপনি আমাদের প্রস্তাবে রাজি হয়ে এই গবেষণায় অংশগ্রহনের সম্মতি প্রদান করেন, তাহলে নিচে আপনার স্বাক্ষর বা টিপসই দিয়ে তা প্রকাশ করুন।
আপনার সহযোগিতার জন্য ধন্যবাদ।

______________________________ ____________________

অংশগ্রহনকারীর অভিভাবক এর স্বাক্ষর বা টিপসই তারিখ

______________________________ ____________________

সাক্ষীর স্বাক্ষর বা টিপসই তারিখ

______________________________ ____________________

প্রধান গবেষক বা তার প্রতিনিধির স্বাক্ষর তারিখ

বিঃদ্রঃ প্রধান গবেষকের প্রতিনিধি তার পূর্ণ নাম ও পদবী লিখে তারপর স্বাক্ষর করবেন।

(আই, আর, বি দপ্তরের যোগাযোগের নাম ও ঠিকানাঃ জনাব এম, এ, সালাম খান, ফোনঃ ৯৮৮৬৪৯৮ বা ৯৮২৭০০১-১০, এক্সটঃ ৩২০৬)
