# Supplementary material for: Injectable Amoxicillin Versus Injectable Ampicillin Plus Gentamicin in the Treatment of Severe Pneumonia in Children Aged 2 to 59 Months: Protocol for an Open-Label Randomized Controlled Trial
Source: JMIR Res Protoc. 2020 Nov 2;9(11):e17735. doi: 10.2196/17735 (PMC7669443; doi:10.2196/17735)
Supplement: Multimedia Appendix 4 [file resprot_v9i11e17735_app4.pdf]

<u>Comments of External Reviewer</u>

Comments from 1st Reviewer (Prof Trevor Duke):

The potential difference between BD Amoxycillin and QID Ampicillin will be if there is true bacterial pneumonia / sepsis. Therefore you need to ensure the study has sufficient proportions of these patients. A cohort of the children with "severe pneumonia" is mixed, with a number of viruses (esp RSV, adenovirus, parainfluenza), viral + secondary bacterial, pneumococcal, Group A Strep, Staphylococcus, TB. Unless the study population has sufficient numbers of bacterial pneumonia, then there is no chance of finding a difference.

So I go back to the definition of severe pneumonia in the study:

Children less than 2 to <59 months will be diagnosed as a case of severe pneumonia if they have a history of cough or difficulty in breathing, plus **at least one of the following**:

i) Central cyanosis or oxygen saturation < 90% on pulse oximetry
ii) Severe respiratory distress (e.g. grunting, very severe chest indrawing)
iii) Signs of pneumonia with a general danger sign: - inability to breastfeed or drink,
- Lethargy or unconscious, convulsions danger signs such as hypoxemia (SO2<90%), cyanosis, grunting respiration, inability to feed, and lethargy.

So a child could have cough or difficult breathing and an SpO2 of 88%. In some populations this would include a lot of infants with bronchiolitis. If the study enrols a large proportion of such children then those with true severe pneumonia will be diluted and the power to see a difference is reduced. I would suggest that the entry criteria are "TWO of the following", rather than one of the following. That would ensure a more severe spectrum of illness among participants, and greater power to see a difference in QID Amp vs BD Amoxyl.

-------- Original message --------
From: Trevor Duke <Trevor.Duke@rch.org.au>
Date:30/05/2017 02:39 (GMT+06:00)
To: Tapon Kumar Bose <tapon@icddrb.org>
Cc: "Dr. Tahmeed Ahmed" <tahmeed@icddrb.org>
Subject: RE: Review of a research proposal

Dear Tahmeed,

I am travelling in PNG at the moment, so I cannot do a full and detailed review. However I have read through the pertinent parts of the proposal. My comments are:

The potential difference between BD Amoxycillin and QID Ampicillin will be if there is true bacterial pneumonia / sepsis. Therefore you need to ensure the study has sufficient proportions of these patients. A cohort of the children with "severe pneumonia" is mixed, with a number of viruses (esp RSV, adenovirus, parainfluenza), viral + secondary bacterial, pneumococcal, Group A Strep, Staphylococcus, TB. Unless the study population has sufficient numbers of bacterial pneumonia, then there is no chance of finding a difference.

So I go back to the definition of severe pneumonia in the study:

Children less than 2 to <59 months will be diagnosed as a case of severe pneumonia if they have a history of cough or difficulty in breathing, plus **at least one of the following**:

i) Central cyanosis or oxygen saturation < 90% on pulse oximetry

ii) Severe respiratory distress (e.g. grunting, very severe chest indrawing)

iii) Signs of pneumonia with a general danger sign: - inability to breastfeed or drink,

- Lethargy or unconscious, convulsions danger signs such as hypoxemia (SO2<90%), cyanosis, grunting respiration, inability to feed, and lethargy.

So a child could have cough or difficult breathing and an SpO2 of 88%. In some populations this would include a lot of infants with bronchiolitis. If the study enrols a large proportion of such children then those with true severe pneumonia will be diluted and the power to see a difference is reduced. I would suggest that the entry criteria are "TWO of the following", rather than one of the following. That would ensure a more severe spectrum of illness among participants, and greater power to see a difference in QID Amp vs BD Amoxyl.

I hope this helps, sorry to not be able to provide a more detailed critique. But I think it could be a very useful study, if the entry criteria are carefully considered.

Kind regards,

Trevor

Review of the project

Title: Efficacy of two doses of parental Amoxicillin plus single dose Gentamycin compared to four doses of parental Amoxicillin plus single dose Gentamycin in managing children 2-59 months of age hospitalized with severe pneumonia: an open labelled randomized controlled clinical trial

Summary of Referee's Opinion: Please see the following table to evaluate the various aspects of the proposal by checking the appropriate boxes. Your detailed comments are sought on a separate attached page.

	Rank score		
	High	Medium	Low
Quality of the project		√	
Adequacy of project design	√		
Suitability of methodology		√	
Feasibility within time period		√	
Appropriateness of budget			
Potential value of field design		√	

CONCLUSIONS

I support the application:

(a) Without qualification
(b) With qualification
- On technical grounds: with modification of title and clarification of methodology
- On level of financial support

I do not support the application

Name of the Referee: Prof ARM Luthful Kabir

Signature : [signature] 6/6/2017

Position :Professor of Pediatrics

Institution: Ad-din Women's Medical College, Bara Magh Bazar, Dhaka

Detailed Comments

The title should be ......WHO classified severe pneumonia instead of ....severe pneumonia only.

The commonest cause of first breathing and chest indrawing in young children is bronchiolitis (21%) and not pneumonia (11.5%) (Kabir ARML, Amin MR, Mollah MAH, Khanam S, Mridha AA, Ahmed S, Rokonuddin K, Chisti J. Respiratory disorders in under-five children attending different hospitals of Bangladesh. *J Respiratory Medicine Research and Treatment 2016, Article ID* 183615, *11 pages)*
and these children improves without any antibiotics (*Kabir ARML*, Mollah A, Anwar KS, Rahman F, Amin R, Rahman ME, Management of bronchiolitis without antibiotics: a multi-centre randomized control trial in Bangladesh. *ActaPaediatrica* 2009; 98 (10): 1593-1599). There is high prevalence of bronchiolitis (59%,

any virus positive 45%) in WHO classified severe pneumonia (Kabir ARML. Uchida R, Ahmed S, Shikder B, Ahmed SU, Khatun S. Predominance of viral bronchiolitis among children with WHO severe pneumonia, *Bronchiolitis compendium, Bangladesh Pediatric Pulmonology Forum 2015*). As there is high chance of improvement without antibiotics, the credit may be imparted to two doses of Amoxicillin or Ampicillin.

It is not clear the dosage of antibiotics during the study. How frequently the Amoxicillin and Ampicillin will be administered not elaborated clearly.

Dhaka hospital is mostly responsible for treating diarrheal diseases (around 110,000 cases each year). It is not clear how many pure WHO classified severe pneumonia cases and how many diarrhea with associated pneumonia are hospitalized into Dhaka Hospital. There might be difference in the outcome between the two groups.

However, this will be a good study in view of reducing the burden of antibiotics in pneumonia cases.
